# Expanding Protected Areas to Safeguard Kenya's Herpetofauna Under Climate Change

**DOI:** 10.1002/ece3.72803

**Published:** 2025-12-22

**Authors:** Ronnie Mwangi Kimani, Chunrong Mi, Patrick Kinyatta Malonza, Beryl A. Bwong, Weiguo Du

**Affiliations:** ^1^ State Key Laboratory of Animal Ecology and Conservation Biology, Institute of Zoology Chinese Academy of Sciences Beijing China; ^2^ University of Chinese Academy of Sciences Beijing China; ^3^ Herpetology Section, Zoology Department National Museums of Kenya Nairobi Kenya; ^4^ Princeton School of Public and International Affairs, Princeton University Princeton New Jersey USA; ^5^ State Key Laboratory of Wetland Conservation and Restoration, National Observations and Research Station for Wetland Ecosystems of the Yangtze Estuary, Ministry of Education Key Laboratory for Biodiversity Science and Ecological Engineering, Institute of Eco‐Chongming, School of Life Sciences Fudan University Shanghai China

**Keywords:** Africa, amphibians, climate change, conservation prioritization, Kenya, protected areas, reptiles, species distribution models

## Abstract

Climate change is a major driver of biodiversity loss, particularly for ectothermic species such as reptiles and amphibians (hereafter herpetofauna), which are highly sensitive to environmental changes. While extensive research has evaluated the effectiveness of protected areas (PAs) in conserving biodiversity under climate change in developed and rapidly developing countries, similar studies in Africa remain scarce despite the continent's exceptional biodiversity. This study focuses on Kenya, home to over 110 amphibians and 290 reptile species, as a model to address this conservation gap in the face of climate change. We used species distribution models (SDMs) to predict herpetofauna distribution for the year 2050 under three Shared Socioeconomic Pathways (SSP1‐2.6, SSP2‐4.5, and SSP5‐8.5). Our results indicate that 11 herpetofauna species (three amphibians with one endemic species and eight reptiles with two being endemic) are at risk of local extinction. Furthermore, over 80% of species in both groups currently have < 30% of their range protected within existing PAs, a trend that persists under future scenarios. We applied a systematic conservation planning approach to address this shortfall to identify priority areas for future conservation efforts. Our findings suggest that Kenya's PA network would need to expand by approximately 16%–19% of the total land area to safeguard herpetofauna both now and in the future effectively. This study underscores the urgent need to optimize Kenya's PA network to mitigate the effects of climate change on herpetofauna. A proactive approach to conservation planning is essential to enhance species resilience and ensure their long‐term survival in a rapidly changing climate.

## Graphical abstract

Climate change threatens herpetofauna species by reducing suitable habitats and limiting species distributions, yet most existing protected areas provide inadequate coverage for them. Using species distribution models and systematic conservation planning, we identified priority areas requiring protected areas expansion to mitigate biodiversity loss in Kenya. Our findings highlight the urgent need to optimize Kenya's protected area network to enhance species resilience and safeguard herpetofauna species against future climate change impacts.

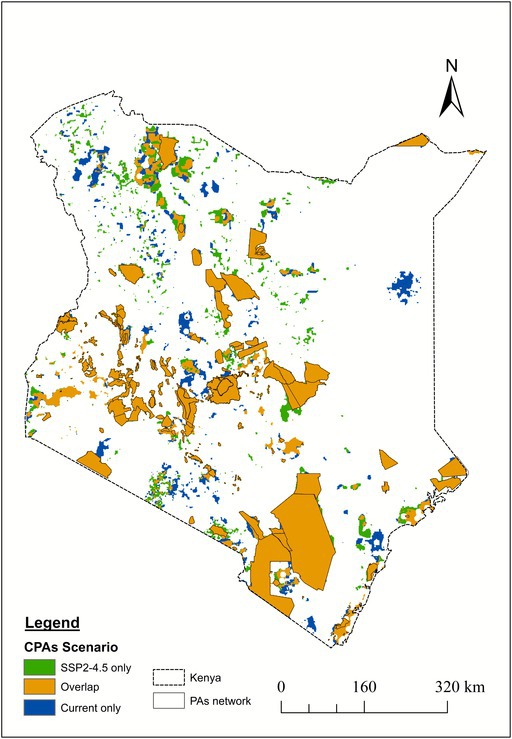

## 1 Introduction

The global emission of greenhouse gases due to human activities has increased the average surface temperature by approximately 1°C over the past century (Calvin et al. 2023). This increase has triggered shifts in climate patterns and a heightened frequency of extreme weather events worldwide (Shivanna 2022). If emissions are not significantly reduced in the coming decades, the Earth's temperature is projected to rise by 1.5°C–2°C by the end of the 21st century (IPCC 2023).

In recent years, anthropogenic pressures, such as agricultural expansion, urban development, climate change, and logging, have contributed to habitat alterations and subsequent biodiversity loss (Harfoot et al. 2021; Farooq et al. 2024). As global warming continues, these trends are expected to intensify, with climate change predicted to drive the future extinction of numerous species (Bellard et al. 2012). There is substantial evidence indicating that anthropogenic climate change is responsible for shifts in species' distribution ranges (Lenoir et al. 2020), widespread population extirpations, such as those documented for lizards (Sinervo et al. 2018), and, consequently, the broader loss of biodiversity across taxa (Cox et al. 2022; Panetta et al. 2018).

As ectotherms, herpetofauna species are highly vulnerable to climate change, as their physiological processes depend on ambient temperature and their capacity to adapt and recover (Huey et al. 2012; Lopez‐Alcaide and Macip‐Ríos 2011). Their distributions are tightly linked to rainfall and temperature patterns (Mi et al. 2024), making them particularly susceptible to climatic shifts that reduce their habitat suitability. Limited dispersal capacity further constrains their ability to track suitable climates, often leading to range contractions and local extinctions rather than expansions (Inman et al. 2022). Globally, over 300 amphibian and 500 reptile species are projected to face extinction from shrinking ranges this century (Mi, Ma, et al. 2023), with more than 41% of amphibians already threatened, climate change being a major driver (de Albuquerque et al. 2024; IUCN 2024; Luedtke et al. 2023). Consequently, herpetofauna serve as valuable indicators for assessing how climate change influences species distributions and the effectiveness of protected areas (PAs).

PAs whose crucial function is the preservation of natural ecosystems through mitigation and elimination of anthropogenic pressures, hence being critical for herpetofauna conservation (Joppa and Pfaff 2010; Nowakowski et al. 2023). However, in many developing countries, PAs often struggle to fulfill this mandate effectively (Fromont et al. 2024; Watson et al. 2014). Such inefficiencies are largely attributed to inadequate funding, weak institutional frameworks, and increasing anthropogenic pressures surrounding PAs. Furthermore, studies by Dobrowski et al. (2021) and Parks et al. (2023) indicate that existing PA networks may be insufficient in protecting global biodiversity under changing climatic conditions despite their potential to serve as climate refugia for herpetofauna species (Mi, Song, et al. 2023). This highlights the need to assess the effectiveness of PAs in conserving biodiversity under localized climate change scenarios, particularly in developing regions such as Africa.

Kenya, situated in the tropical region of Africa, harbors a rich diversity of herpetofauna, with over 110 amphibian and 290 reptile species recorded (Malonza and Bwong 2023). The primary threats to these species are driven mainly by habitat loss and deforestation, with approximately 15% of Kenya's herpetofauna classified as threatened (IUCN 2024). For instance, *Arthroleptides dutoiti* , an amphibian species endemic to Mount Elgon, has become locally extinct on the Kenyan slopes of the mountain (Ngwava et al. 2021), with climate change being implicated in loss of amphibian species (Lötters et al. 2023). There are geographical biases and scarcity in studies focusing on the effects of climate change on herpetofauna (Tan et al. 2023). In Kenya, research on the impacts of climate change on herpetofauna species is minimal. Notably, species previously recorded in arid regions are now being observed in areas historically classified as wet forested highlands, suggesting shifts in distribution due to warming temperatures (Malonza and Bwong 2023). Given these changes, further studies are needed to assess the effects of climate change on Kenya's herpetofauna at a macro scale.

This study sought to understand how climate change will impact the suitable habitats of herpetofauna at present and under future climate change scenarios in Kenya. It aims to provide decision‐makers with comprehensive data and assessments to protect and conserve the underrepresented herpetofauna species and their habitats in Kenya. Precisely, our study employed species distribution modeling to (1) predict species distributions under future climatic conditions, (2) quantify the representation of herpetofauna species in PAs, and (3) identify nationwide conservation priority areas (CPAs) that ensure effective conservation of herpetofauna in Kenya under climate change scenarios.

## 2 Materials and Methods

### 2.1 Occurrence Records

Occurrence data for herpetofauna in Kenya were collected using a multistep approach. First, we used the latest comprehensive field guide on Kenyan herpetofauna (Malonza and Bwong 2023). Using Google Earth Pro (V 7.3), we geotagged the local names of the distribution locations provided by the field guide. Second, we extracted published literature from Web of Science and Google Scholar by searching with keywords such as “Species Distribution Models,” “Species richness,” and “Species diversity.” The results were filtered for “Kenya” and/or “Amphibians” or “Reptiles” and limited to articles published between 2000 and 2023. Third, to complement the data collected, we compiled data from online repositories, including GBIF, VertNet, and iDigBio.

After compiling the data, occurrence records were cleaned using the “CoordinateCleaner” package (Zizka et al. 2019) to eliminate records located in cities, institutes, and museums with a buffer of 10 km, and 100m for both respectively. The “spThin” package in R was then applied to remove duplicate occurrences within a single 1 km × 1 km grid cell and to exclude species with fewer than five records, thereby reducing sampling biases (Aiello‐Lammens et al. 2015). The cleaned dataset was subsequently visualized in ArcGis to display the distribution of herpetofauna in Kenya, and the clip function was used to remove records outside Kenya's administrative boundaries. This process yielded 2329 occurrence records representing 97 (~88%) amphibian species and 6948 records representing 243 (~84%) reptile species, which were fed to the species distribution models.

### 2.2 Environmental Variables

Environmental variables representing current (1970–2000) and future (2041–2070) climatic conditions were obtained from the “WorldClim” database (https://www.worldclim.org/). To address uncertainties in future projections (hereafter referred to as 2050), the raster data of five Global Circulation Models—GFDL‐ESM4, IPSL‐CM6A‐LR, MPI‐ESM1‐2‐HR, MRI‐ESM2‐0, and UKESM1‐0‐LL—were averaged. This dataset includes 19 bioclimatic variables (BIO1–BIO19) at a 30‐s spatial resolution. Additionally, three Shared Socioeconomic Pathways (SSP1‐2.6, SSP2‐4.5, and SSP5‐8.5) were considered while predicting future distributions. The environmental layers were reprojected to a Lambert Azimuthal Equal Area projection (Herkt et al. 2017), centered at 10° N, 38° E, with a grid cell resolution of 1 km × 1 km.

### 2.3 Species Distribution Modeling

To develop the SDMs, we used the “sdm” package in R statistical software (Naimi and Araújo 2016). We employed an ensemble approach combining five widely used algorithms: Random Forest (RF), Generalized Linear Model (GLM), Maximum Entropy (MAXENT), Support Vector Machines (SVM), and Generalized Boosted Regression Models (GBR) (Elith and Leathwick 2009; Naimi and Araújo 2016). Pseudo‐absence data were generated by randomly assigning points in unoccupied grid cells following the methods of Mi, Ma, et al. (2023). We applied the “vif” function from the “usdm” R package (V 2.1–7) to minimize multicollinearity, filtering out bioclimatic variables with a variance inflation factor (VIF) > 10 (Naimi et al. 2014). After accounting for multicollinearity, 10 bioclimatic variables were retained for species distribution modeling (BIO 2, 4, 7, 9, 12, 13, 14, 15, 18, and 19). The mean and standard deviation of these variables across all species are presented in Table S1. The dataset was randomly split, with 80% allocated for model training and the remaining 20% used for cross‐validation. Models with a True Skill Statistic (TSS) value ≥ 0.7 were retained for the final ensemble model (Gallardo et al. 2017). Model performance was evaluated using the Area Under the Curve (AUC) and TSS. We then converted the habitat suitability maps into binary presence‐absence maps using the Max‐TSS threshold (Gallardo et al. 2017; Liu et al. 2016). Given the limited dispersal capabilities of most herpetofauna species, additional analyses were conducted under a no‐dispersal assumption (Smith and Green 2005).

### 2.4 Estimating Species Richness and Rarity‐Weighted Richness

The binary SDM maps generated for each species were used to estimate species richness under current and future climate scenarios. Species richness was calculated using the calc function from the raster package in R, determining the number of species present in each grid cell for both timeframes. Although raster has been replaced by Terra, it was used in this study for compatibility with other modeling packages integrated into the workflow. Differences in species richness between current and future projections were then analyzed to assess the impact of climate change on suitable habitats for herpetofauna species.

Rarity‐weighted richness (RWR) was calculated following the methodology outlined by de Albuquerque and Gregory (2017). This process involved two steps: First, we assigned a rarity score to each species, calculated as the inverse of the number of grid cells a species occupies. For instance, a species restricted to a single grid cell received the maximum score (1.0), while a species occurring in 100 grid cells received a lower score (0.01, calculated as 1/100). Second, the rarity scores for each species were then summed across grid cells using the formula:

$$\mathrm{RWR}=\sum_{1}^{n}\frac{1}{c_{i}}$$

where *c*_*i*_ represents the number of grid cells occupied by species *i*, which is summed for the *n* species occurring in a given grid cell. RWR was calculated for current and future scenarios to evaluate the potential impact of climate change on the rarity‐weighted richness of herpetofauna species.

### 2.5 Range of Herpetofauna Inside PAs

To assess the effectiveness of PAs, geospatial data for PAs in Kenya were obtained from the World Database on Protected Areas (WPDA) (https://www.protectedplanet.net/en). PAs classified under IUCN management categories I–VI were considered, excluding marine PAs. The projected distribution maps were first reprojected into the Lambert Azimuthal Equal Area projection to calculate species range sizes. We calculated each species' range size inside PAs using the binary maps for current and future scenarios. The species range sizes inside PAs were then categorized according to the following categories to show the representation of amphibians and reptiles inside the existing PAs network: unprotected (UP) ≤ 10%; inadequately protected (IP) > 10% ≤ 30%; partially protected (PP) > 30% ≤ 50%; adequately protected > 50% ≤ 80%; protected > 80%. These thresholds were selected to provide a clear and interpretable framework for assessing species representation inside Kenyan PA (Barata et al. 2016). To evaluate the future effectiveness of the current PA network in conserving herpetofauna diversity, we used the projected range size maps for both current and future scenarios, calculating the percentage of species falling within each of the five representation categories.

### 2.6 Conservation Prioritization

To optimize the existing PAs network in Kenya for the effective conservation of the 340 herpetofauna species, we used the Marxan software (Ball et al. 2009), a conservation planning tool designed to optimize resource allocation for biodiversity preservation while minimizing costs. The planning limits were set using Kenya's administrative boundary, and planning units of 1 km^2^ were created, resulting in a total of 588,327 planning units. Each planning unit was assigned a status code prior to running Marxan. For consistency in GIS preprocessing, we re‐coded the planning units as follows: status = 1 (“locked in”) for those overlapping with existing protected areas; status = 2 (“locked out”) for those within a 5 km buffer of towns, municipalities, or city centers; and status = 0 (“available”) for the remaining units. In Marxan, these codes were implemented with the same functional meaning as the standard input convention, that is, locked‐in units always included, locked‐out units always excluded, and available units selectable in the solution. The cost estimates for each planning unit were derived from the Human Footprint Index (Venter et al. 2016), calculated by incorporating data on human pressures, such as population density, built environments, and land use, which compete with biodiversity conservation, thus providing a reliable baseline for cost.

We incorporated the binary range maps for the 340 herpetofauna species generated by the SDMs, resulting in a matrix of planning units categorized by conservation features. Extensive literature has discussed the minimum protection required to ensure species' long‐term survival. One standard method is setting conservation targets based on species range size, which varies depending on geographic distribution. We adopted the criteria from Carwardine, Wilson, Ceballos, et al. (2008), Carwardine, Wilson, Watts, et al. (2008), and Mi, Ma, et al. (2023), which apply tiered conservation targets analogous to the logarithmic decay function originally proposed by Rodrigues et al. (2004): if a species' predicted range is ≤ 1000 km^2^, the target is set at 100%; for ranges between ≥ 1000 and ≤ 10,000 km^2^, the target is 30%; and for species with ranges > 10,000 km^2^, the conservation target was set at 10%. The range maps used were restricted within the Kenyan boundary.

We ran four independent spatial prioritization scenarios to explore optimal conservation solutions for Kenyan herpetofauna under current and future scenarios. Each scenario was executed with 1000 repetitions, constituting 10 million iterations. The “best solution” output from Marxan was selected as the final solution for the CPAs. We performed a cluster and outlier analysis in ArcGIS (Anselin Local Moran's *I*) using Euclidian distance as the similarity metric to identify planning units with high conservation values. The method identifies statistically significant high value and low value clusters as well as high‐low and low‐high outliers. We then overlapped the current and future CPAs to identify regions selected in both scenarios. The boundary length modifier was calibrated using the ArcMarxan Toolbox, with a value of ~0.001 for each scenario. ArcGIS 10.8 was used to create the planning units and assign their statuses. At the same time, R software (Version R‐4.3.2) (R Core Team 2023) was employed to calculate each planning unit's cost, feature value, and conservation target for each species.

## 3 Results

### 3.1 SDM Performance

Our models performed well for all 340 herpetofauna species included in the analysis (AUC = 0.951 ± 0.034, TSS = 0.883 ± 0.085; see Table S5). This performance is highlighted by our prediction that 11 herpetofauna species will experience local extinction, losing all their suitable habitats, under the SSP2‐4.5 scenario by 2050 (see Table S2 for additional scenarios and the endemicity of the locally extinct species). Our ensemble models also revealed that, under the current scenario, four amphibian and five reptile species have suitable habitats entirely outside the existing PAs network. This number increases to seven reptile and four amphibian species under the SSP2‐4.5 scenario (see Table S3 for other scenarios).

### 3.2 Species Richness

**FIGURE 1:**
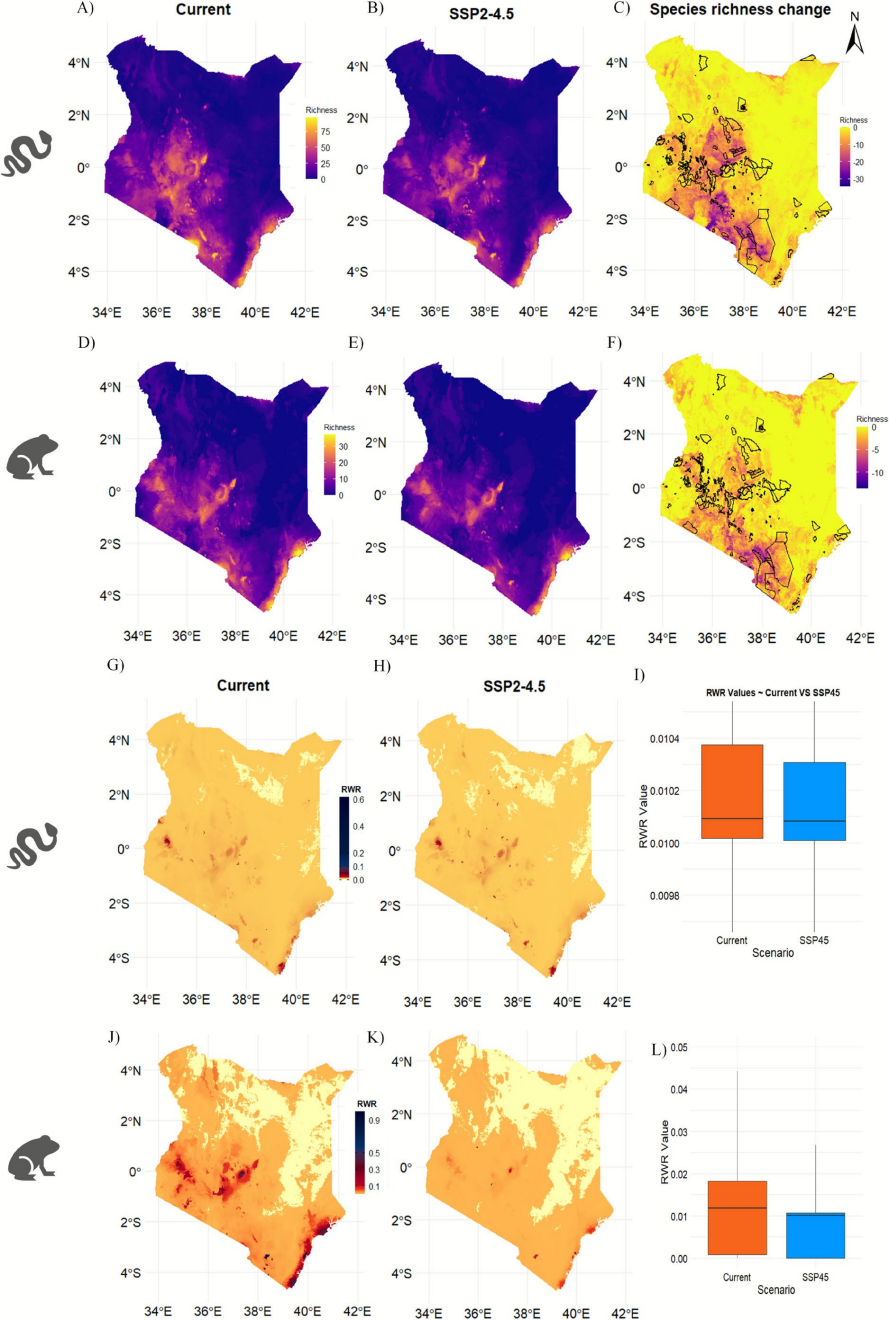
(A—F) Species richness of Kenyan herpetofauna for current and future scenarios. The 1st and 2nd columns show species richness and RWR for current and future scenarios, while the 3rd column shows the change in species richness in the first two rows (C, F). (G, H, J, K) RWR for reptiles and amphibians under current and future SSP2‐4.5 climatic scenarios by 2050. The color scale indicates lower values in yellow and higher values in dark red. (I, L) Box plots showing the difference between current and SSP2‐4.5 RWR values (Wilcoxon test; *Z* = 73.8, *p* value < 0.001, and *Z* = 389, *p* value < 0.001) for I and L, respectively. The box plots were zoomed in while plotting because more RWR values were < 0.02, causing the box plots to be squeezed in.

Our SDMs predicted similar distribution patterns for both reptile and amphibian species in Kenya (Pearson correlation: *r* = 0.881, *p* < 0.001). The current scenario shows that species richness is concentrated in the central, western, and southeastern regions and along the humid coastal belt. Similar distribution patterns were observed in future predictions (Pearson correlation: *r* = 0.968 for amphibians, *r* = 0.966 for reptiles), with these regions also facing habitat loss (see Figure 1C, Figures S1 and S2 for other scenarios). In contrast, the northern and eastern areas exhibited lower species richness. Similar species richness patterns were observed among the different taxa group of reptiles as seen in Figures S14–S25 for all the future climate scenarios. Our maps indicated regions experiencing significant species richness loss were predominantly outside the existing PAs (Figure 1C,F). However, results from the Wilcoxon test showed no significant difference in proportional range loss between areas located inside and outside PAs (see Figures S9–S11). Notably, the interquartile range for proportional range loss in areas inside protected areas was slightly lower for both taxa across all scenarios.

### 3.3 Rarity‐Weighted Richness

Our analysis revealed that high RWR regions (RWR > 0.3) for both taxa were concentrated along the coastal strip, Taita Hills toward the Eastern Arc Mountains (southern part of the country), the western region near Kakamega Forest, Nandi Hills, through Mau Forest to the Aberdare Ranges, and the slopes of Mount Kenya (central region of the country) (Figure 1). In the future, some reptile species are predicted to become rare as regions with relatively high RWR values emerge. For amphibians, we observed a reduction in areas with high RWR values. This was evident as both taxa exhibited narrower interquartile ranges (Q3) in the boxplots of all future scenarios compared to the current scenario (Figure 1I,L for SSP2‐4.5 and Figures S3 and S4). In all scenarios, regions along the Taita Hills displayed high RWR values for both taxonomic groups. However, they do not fall within any of the four IUCN management categories (IUCN I–VI) of PAs.

### 3.4 Effectiveness of Current PAs and Proposed CPAs

**FIGURE 2:**
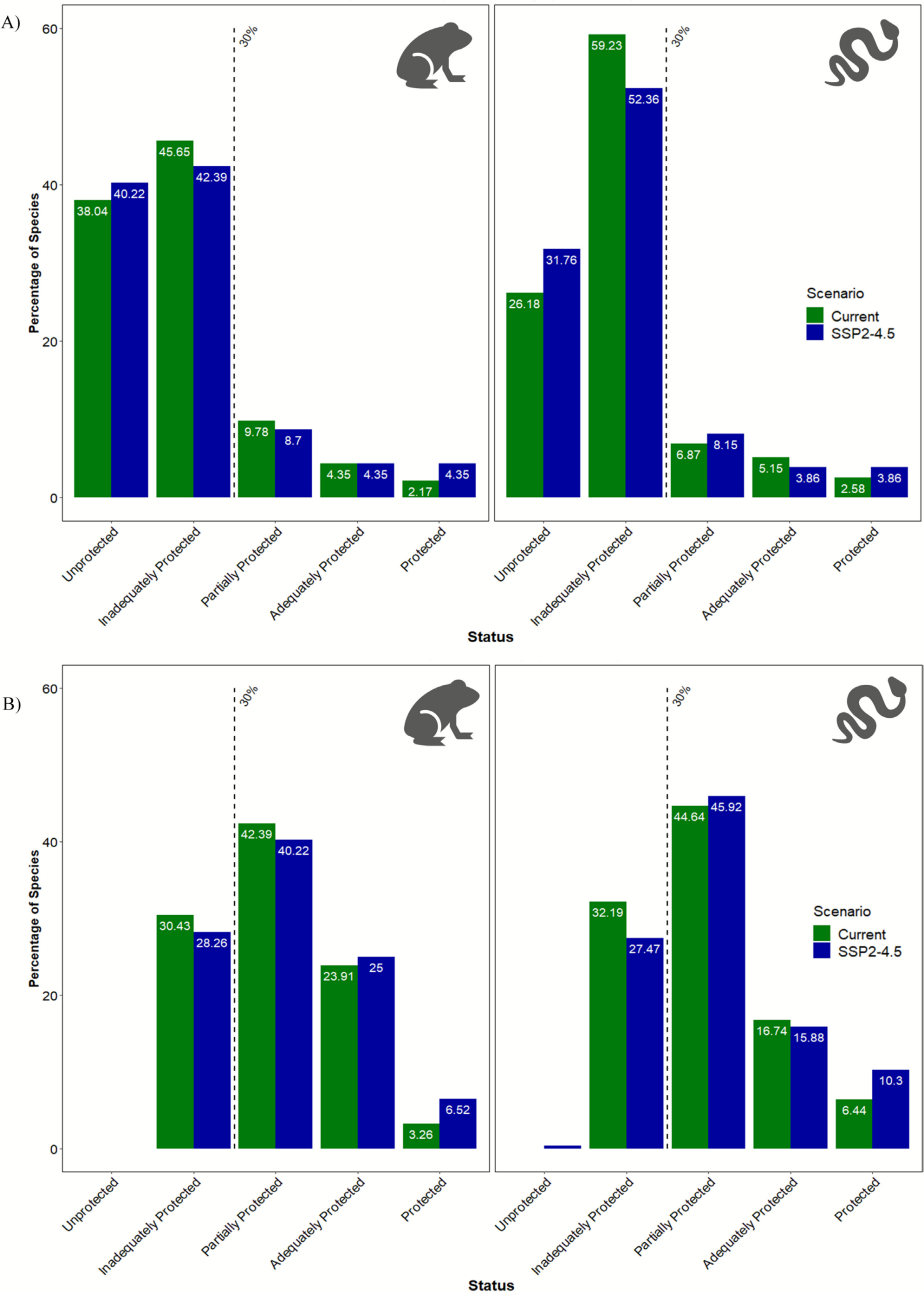
The graph shows the proportion of herpetofauna species protected by (A) the current PAs network and (B) Marxan's CPAs network for Reptiles and Amphibians for the four‐conservation status under SSP2‐4.5 by 2050.

In the current scenario, approximately 38% of amphibian species fall under the “Unprotected” (≤ 10%) category, a proportion that increases to about 40% by 2050. For reptiles, this proportion increases from around 26% to approximately 31% (see Figure 2, Figures S12 and S13). The percentage of species in the “Inadequately protected” category (≤ 30%) decreased for both groups: from approximately 45% to 42% for amphibians and from 59% to 52% for reptiles. The “Protected” category also saw a modest increase, with amphibians rising from 3.1% to 4.1% and reptiles from 3.3% to 3.7% under the SSP2‐4.5 scenario. An increase in the percentage of species in the “Protected” category was also observed in the other scenarios (see Figures S12 and S13).

Our findings indicated that, currently, four (one endemic) amphibian species and seven (two endemic) reptile species have their range sizes entirely outside any form of protection (Table S3). In the future, this number is projected to rise to seven (two endemic) amphibians and 16 (four endemic) reptiles (Table S3). No significant difference was observed in the relative range change of both amphibians and reptiles inside versus outside protected areas (Wilcox test; *p* = 0.93, *Z* = 0.08 for amphibians; *p* = 0.78, *Z* = 0.28 for reptiles; see Figures S9–S11 for other two scenarios).

### 3.5 Spatial Optimization of Existing PAs

**FIGURE 3:**
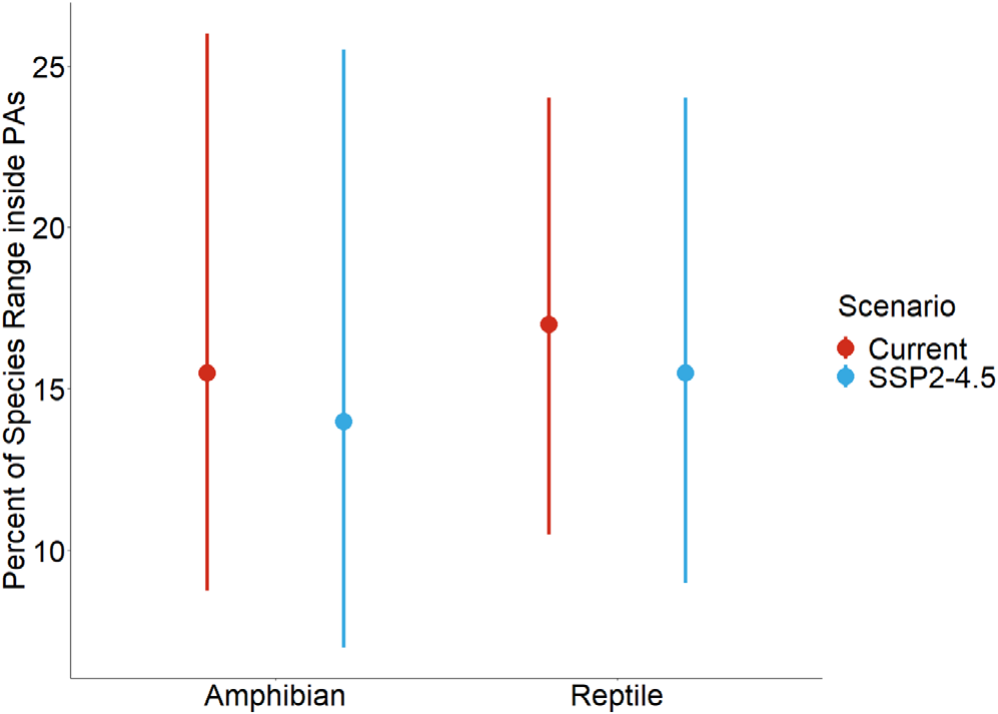
Inter‐quantile range (q1, median, and q3) of the percentage of species range distributed inside existing PAs. “Red” is for the current scenario, and “blue” is for the SSP2‐4.5 scenario by 2050.

A total of 83% of amphibian species and 85% of reptile species had < 30% of their range size within PAs. Across all future scenarios, the proportion of species' range sizes represented within existing PAs declined (see Figure 3; Figures S5 and S6). Currently, PAs cover approximately 10.2% of Kenya's total area (63,247 km^2^). Optimization with Marxan increased this coverage to 16.4% for current conservation priority areas (CPAs) and 16.2% for future CPAs (Table S4).

**FIGURE 4:**
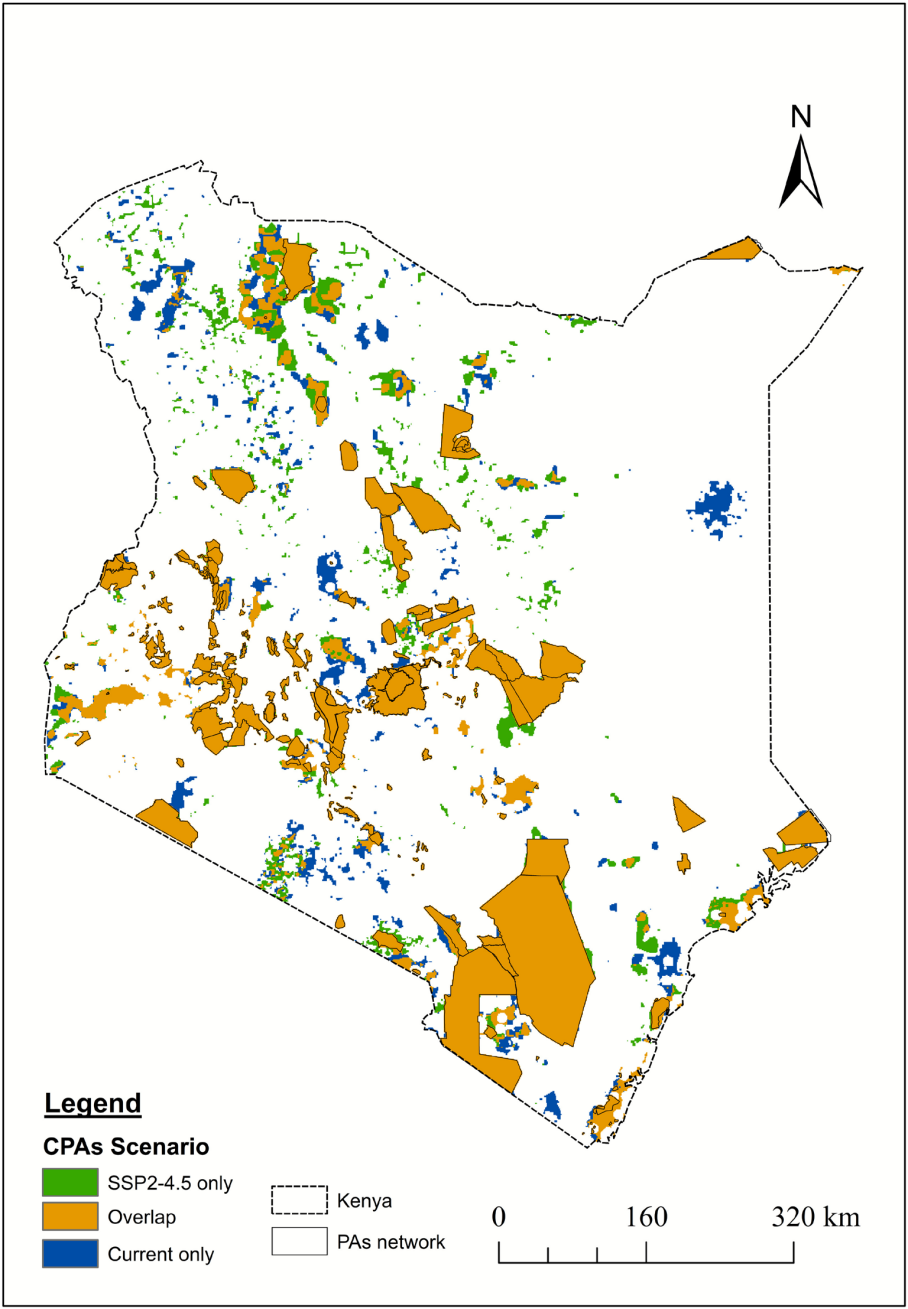
CPAs proposed by spatial prioritization analysis.

We identified several “overlapped” regions—areas selected as CPAs in both current and future scenarios—including the Lower Tana River delta, Shimba Hills National Reserve, the coastal belt, and the Lake Victoria basin. In the current scenario, additional CPAs were proposed along the eastern and northwestern borders, Laikipia, the Aberdare slopes, and the Mount Kenya region. In contrast, future additional CPAs included areas along the Lake Turkana region, Arabuko Sokoke Forest, and Kajiado County. Notably, regions along the coastal, southern, and northwestern areas, such as those surrounding Sibiloi National Park, were consistently selected as CPAs across all future scenarios, highlighting their importance for herpetofauna conservation (see Figure 4, Figures S7 and S8).

Our spatial prioritization analysis demonstrated improved herpetofauna representation within the proposed CPAs. The proportion of species with < 30% of their relative range size inside CPAs decreased to below 30% in both current and future scenarios. Furthermore, the percentage of species in the “Unprotected” (≤ 10%) category was reduced to 0% across all future scenarios. Cumulatively, the proportion of species classified as “Partially Protected,” “Adequately Protected,” and “Protected” increased significantly—from 14.6% and 16.3% to 67.82% and 69.56% for reptiles and amphibians, respectively, in the current scenario, and from 15.87% and 17.4% to 70.36% and 69.23% under SSP2‐4.5 (see Figure 2, Figures S12 and S13).

## 4 Discussion

Our study revealed high herpetofauna species richness and RWR to be distributed across Kenya's central, western, and coastal regions. Future projections indicate that many areas at risk of biodiversity loss fall outside the existing PA network, with over seven species expected to lose all their suitable habitats. Our findings also highlight the inefficacy of Kenya's current PAs in protecting herpetofauna, both now and in the future, as more than 80% of species have < 30% of their range within these protected boundaries. To address this conservation gap, we used Marxan analysis to identify CPAs, which reduced the proportion of species with < 30% range protection to below 35%.

Our results underscore the inadequacy of the existing PA network in safeguarding herpetofauna under current and future climate change scenarios. A significant number of species fall into the “Unprotected” category (< 10% of their range within PAs) or the “Inadequately Protected” category (10%–30% intersection), and this trend persists across future projections (see Figure 2, Figures S12 and S13). This indicates that many species remain vulnerable as climate change further restricts their suitable habitats. We found no significant difference in range loss inside versus outside PAs. This suggests that the current PA network does not align with predicted range shifts and is ineffective as climate refugia for herpetofauna. This may also imply that existing PAs do not provide sufficient climate‐buffering effects for herpetofauna species.

We also identified a critical conservation gap, as four amphibian species (one endemic) and five reptile species (one endemic) currently have their entire ranges outside Kenya's existing PAs, with this number projected to increase under future climate scenarios (Table S3). These species face heightened extinction risks due to the absence of alternative habitats within protected areas (Işik 2011). Endemic species are particularly vulnerable, as their restricted ranges limit their access to suitable microhabitats and potential climate refugia. Limited dispersal ability further constrains their capacity to move in response to environmental change, increasing the likelihood of local extinction. Our findings reinforce concerns about the inadequacy of Kenya's current PA network in protecting herpetofauna. This aligns with previous studies indicating that only 5% of the critically endangered Pancake tortoise population occurs within existing PAs (Eustace et al. 2021). Similarly, low representation within PAs has been observed for other vertebrate species in Kenya (Ogutu et al. 2016; Onditi et al. 2021; Tyrrell et al. 2020), as well as for African lions (Robson et al. 2022) and mammal populations in other global regions (Juárez‐Ramírez et al. 2025; Williams et al. 2022), underscoring the urgent need for PA expansion to mitigate biodiversity loss.

Our analysis revealed that several species falling entirely outside the existing PA network are currently listed by IUCN Red List as Vulnerable and Endangered species (see Table S3). This overlap highlights a critical conservation gap, where species most at risk of extinction remain unprotected by the formal PA system. The absence of these threatened species within PAs suggests that current networks may not adequately protect Kenyan herpetofauna species. In addition, species projected to be locally extinct under future climatic conditions include several herpetofauna species that are already considered threatened under current conditions (see Table S2). This underscores the threats Kenyan herpetofauna species are facing, emphasizing the urgent need to reassess PA boundaries to account for their conservation needs.

Using Marxan for spatial prioritization, we identified key areas that would increase the overall area of PAs to approximately 16.4% under current and future SSP2‐4.5 scenarios (Table S4). Marxan consistently selected critical regions as CPAs across all scenarios, including the Lower Tana River, Shimba Hills, the Lake Victoria basin, and areas around Kitobo Forest in Tsavo West National Park (Figure 4, Figures S7 and S8). These regions play a crucial ecological role for Kenyan herpetofauna and could serve as essential refugia against climate change. Expanding PAs to encompass these areas would help establish a climate‐resilient network, enhancing long‐term species survival. Additionally, new CPAs were identified in northern Kenya (near Lake Turkana), along the coastal belt (around Arabuko Sokoke), and in the southern regions (Kajiado near Mount Kilimanjaro). These areas are currently underrepresented in the PA network but are critical for herpetofauna conservation as species range shifts in response to climate change. Notably, Sibiloi National Park is the only existing PA in northwestern Kenya, yet the region has been identified as vital for herpetofauna conservation in future scenarios, emphasizing the need for proactive conservation planning to safeguard these habitats.

Our findings emphasize the necessity of expanding existing PAs to address conservation gaps for Kenyan herpetofauna and enhance their resilience to habitat loss. Specifically, our analysis suggests extending Maasai Mara, Tsavo East and West National Parks, Mount Kenya National Park, and Mwingi National Reserve to incorporate species ranges currently outside their boundaries. Such expansions would improve habitat connectivity, facilitating species dispersal and genetic flow in alignment with global conservation strategies (Beger et al. 2022). For instance, expanding Maasai Mara could enhance connectivity with Chepalungu Forest Reserve, benefiting herpetofauna and other vertebrates responding to climate pressures. Scientific consensus supports the expansion of PA networks as a means to preserve natural ecosystems, safeguard biodiversity, and enhance ecosystem services (Dinerstein et al. 2019; Watson et al. 2020). Additionally, our study highlights that small and isolated CPAs identified through Marxan spatial analysis will serve as climate refugia in future scenarios, making them essential for the conservation of Kenyan herpetofauna under climate change. The importance of such micro‐reserves has been emphasized in recent research (Drechsler et al. 2020; Steigerwald et al. 2024), reinforcing the need to integrate them into Kenya's conservation strategy, particularly in climate‐sensitive regions of eastern Kenya.

Our study mapped similar hotspots of RWR and species richness for Kenyan herpetofauna, particularly in the central, western, and coastal regions. These areas are characterized by diverse vegetation, complex topography, and favorable microhabitats, which support high species richness and endemism. The overall similarity in amphibian and reptile distribution patterns likely reflects the influence of Kenya's geographic and climatic gradients, which shape habitat suitability across taxa. However, projections indicate that these regions could experience significant species loss by 2050 due to climate change (Figure 1, Figures S3 and S4). Narrower interquartile ranges for projected future RWR values suggest that regions with high RWR may contract as species' ranges shrink, leading to a decline in range‐restricted species within their habitats. The potential loss of rare species could contribute to increased species homogenization in these areas (Montràs‐Janer et al. 2024). By identifying regions at risk of species loss, our study provides valuable insights for targeted conservation efforts. Endemic species are particularly vulnerable, with our analysis indicating that four herpetofauna species may face extinction under various scenarios (Table S2). Many of these species are already threatened by anthropogenic pressures, and climate change will likely exacerbate their risk of extinction. A notable example is *Ancylodactylus mathewsensis*, endemic to the hilltop montane forests of the Mathews Range, which is already experiencing habitat modification due to human activities (de Jong and Butynski 2010). This underscores the urgent need for targeted conservation actions within CPAs to mitigate biodiversity decline and maintain ecosystem resilience (Valiente‐Banuet et al. 2015). However, further work separating sub‐groups (e.g., lizards, snakes, turtles, frogs, and toads) is needed to evaluate the role of taxon‐specific traits in structuring richness patterns.

Kenya is committed to the Kunming‐Montreal Global Biodiversity Framework, which aims to protect 30% of terrestrial and marine areas by 2030. However, human and financial constraints limit conservation efforts, highlighting the need for systematic planning in conservation strategies. Protected areas are often established without explicitly incorporating conservation objectives, mainly due to land acquisition challenges (Rodrigues et al. 2004). As a result, many PAs are designated in marginal lands with lower conversion pressures, limited biodiversity importance, or low conservation urgency. Instead, countries should assess whether designated PAs align with the highest conservation priorities before their establishment (Eckert et al. 2023). Kenya is not exempt from this spatial mismatch, as most government‐managed national parks were initially converted from game‐hunting reserves and primarily established to protect large, tourist‐attraction mammals such as elephants, lions, rhinos, buffalos, and leopards (Caro 2003). However, there has been a global shift toward systematic conservation planning (Kareiva et al. 2014), which aims to achieve cost‐effective and strategic PA establishment by identifying biodiversity hotspots for conservation prioritization (Carwardine, Wilson, Ceballos, et al. 2008; Carwardine, Wilson, Watts, et al. 2008). Systematic planning has proven to be an effective approach for identifying CPAs, as it explicitly seeks to meet conservation goals by representing a broad range of biodiversity features, ensuring their long‐term persistence. To enhance the effectiveness of Kenya's PA network in conserving herpetofauna, expansion and prioritization should focus on regions with high herpetofauna endemism (Rodrigues and Gaston 2001).

## 5 Conclusion

Our findings emphasize the significant impact of climate change on Kenyan herpetofauna and the urgent need for targeted conservation efforts. Climate change is reducing suitable habitats and contracting species' ranges, exacerbating their vulnerability. Additionally, our study identified a critical conservation gap in the representation of herpetofauna within existing PAs, both now and in future climate scenarios. These findings underscore the need for systematic conservation planning to enhance species resilience and mitigate biodiversity loss.

While our study provides robust conclusions based on climatic variables, it has some limitations. We did not incorporate other biotic factors (e.g., species‐specific traits, biotic interactions) or abiotic variables (e.g., land use and land cover change) in our analysis. This omission may have led to an underestimation of climate change impacts on the distribution of Kenyan herpetofauna and their integration into conservation strategies. Future research should address these gaps by developing mechanistic models to understand herpetofauna responses to climate change. Despite these limitations, our study offers critical insights into adaptive and targeted conservation strategies for Kenyan herpetofauna in the face of a rapidly changing climate.

## Author Contributions

**Ronnie Mwangi Kimani:** data curation (lead), formal analysis (lead), investigation (lead), methodology (equal), software (equal), visualization (lead), writing – original draft (lead), writing – review and editing (equal). **Chunrong Mi:** conceptualization (supporting), methodology (equal), software (equal), supervision (supporting), writing – review and editing (equal). **Patrick Kinyatta Malonza:** conceptualization (supporting), writing – review and editing (equal). **Beryl A. Bwong:** writing – review and editing (equal). **Weiguo Du:** conceptualization (lead), funding acquisition (lead), supervision (lead), writing – review and editing (equal).

## Funding

This research is funded by the National Natural Science Foundation of China (32330067, 32030013, 32300420 and 32311530110). Ronnie Mwangi Kimani was supported by the ANSO Scholarship for Young Talents (no. 2021ANSOM013). Chunrong Mi was supported by National Key Research Development Program of China (2022YFF0802300).

## Conflicts of Interest

The authors declare no conflicts of interest.

## Supporting information


**Appendix S1:** ece372803‐sup‐0001‐AppendixS1.docx.

## Data Availability

All data and codes used to run the SDMs and range size data generated to create Figures 2 and 3 can be found at https://figshare.com/s/1b970870a64d4c07da23. Climate data were downloaded from the WorldClim database (www.worldclim.org). Shapefiles for Kenyan Protected areas were downloaded from the World Database on Protected Areas (WPDA) (www.protectedplanet.net). The main text and Supporting Information present all other data as figures.
