# Supplementary material for: Expanding Protected Areas to Safeguard Kenya's Herpetofauna Under Climate Change
Source: Ecol Evol. 2025 Dec 22;15(12):e72803. doi: 10.1002/ece3.72803 (PMC12720015; doi:10.1002/ece3.72803)
Supplement: Supplementary file 1 — Appendix S1: ece372803‐sup‐0001‐AppendixS1.docx. [file ECE3-15-e72803-s001.docx]

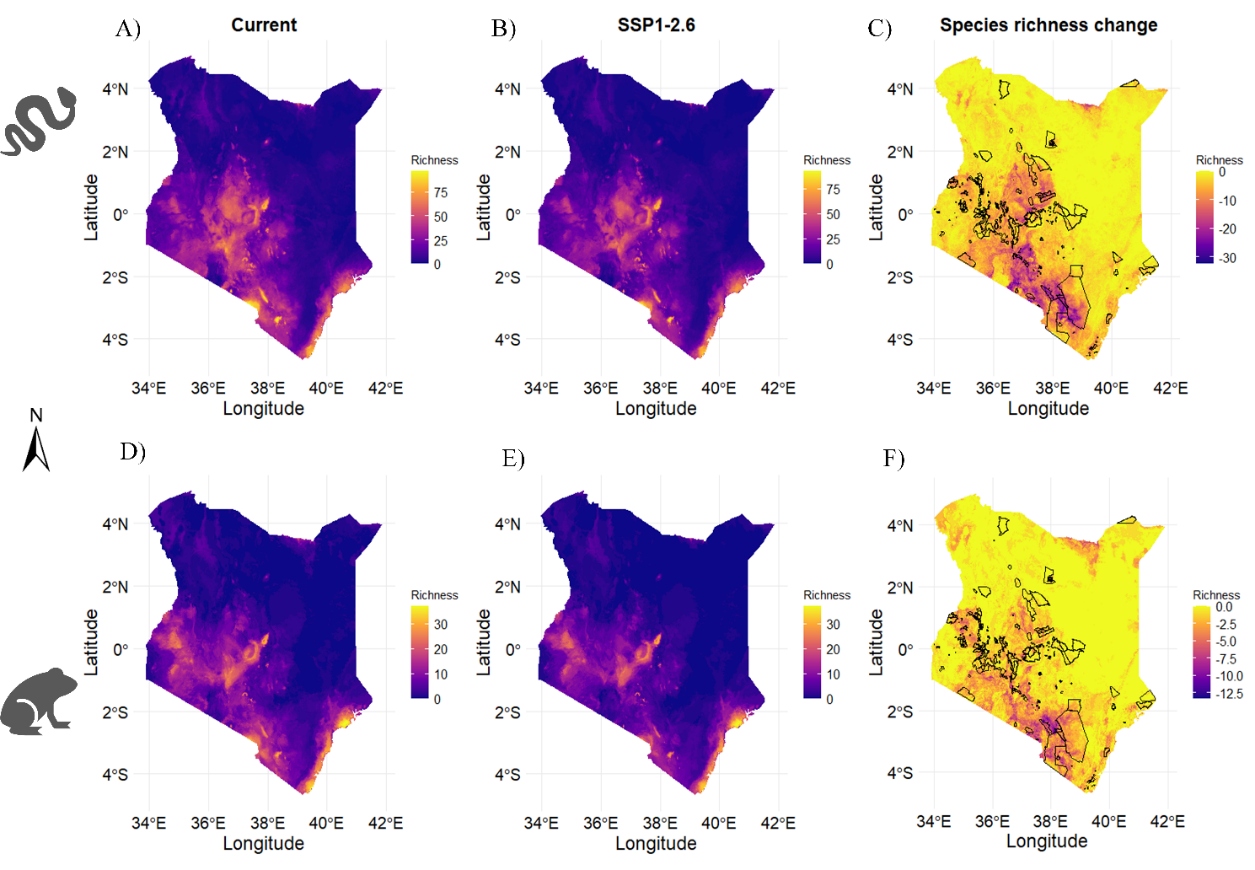


**Figure S1 Species richness and change in richness of Kenyan herpetofauna species for current and SSP1-2.6 scenarios**

**NB:** Polygons in 3^rd^ column represent the current PA network in Kenya.


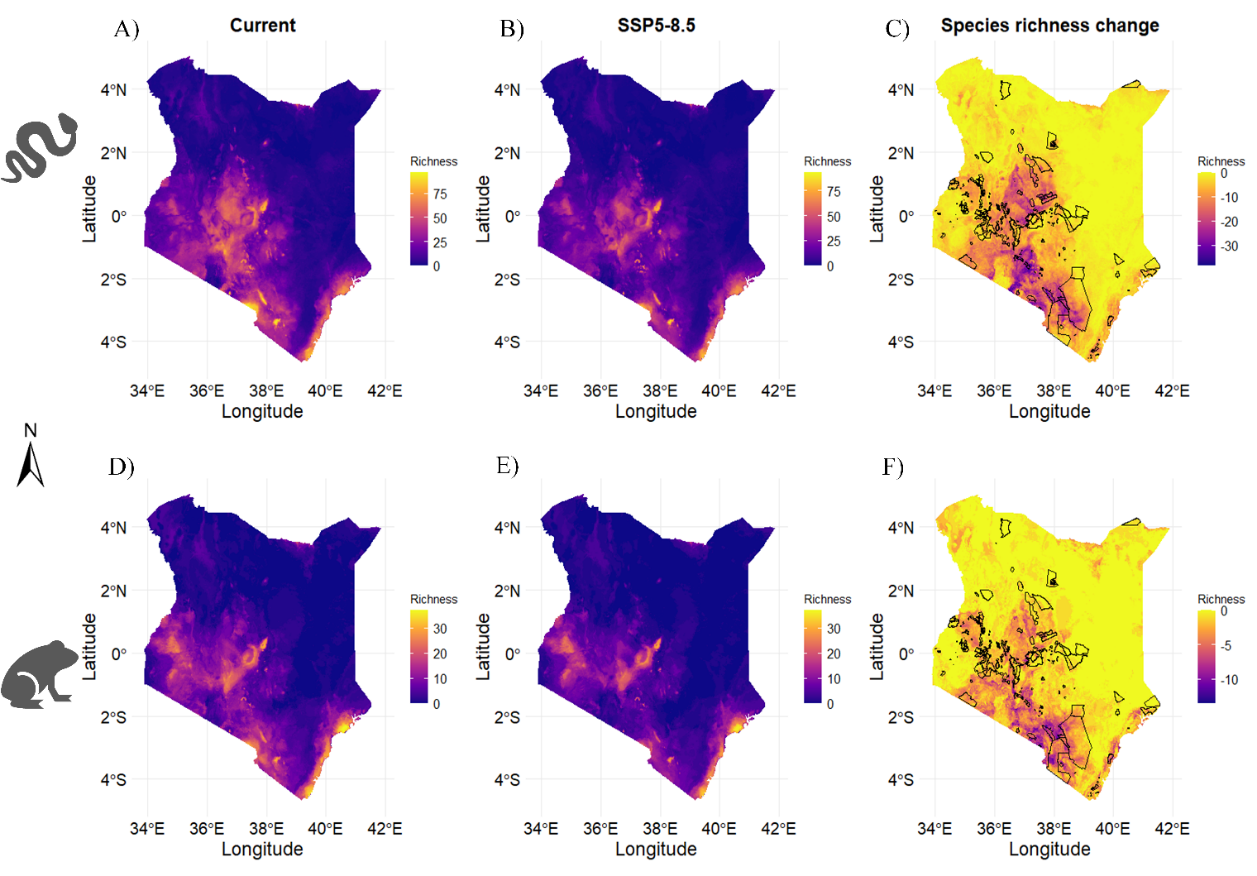


**Figure S2 Species richness and change in richness of Kenyan herpetofauna species for current and SSP5-8.5 scenarios**

**NB:** Polygons in 3^rd^ column represent the current PA network in Kenya


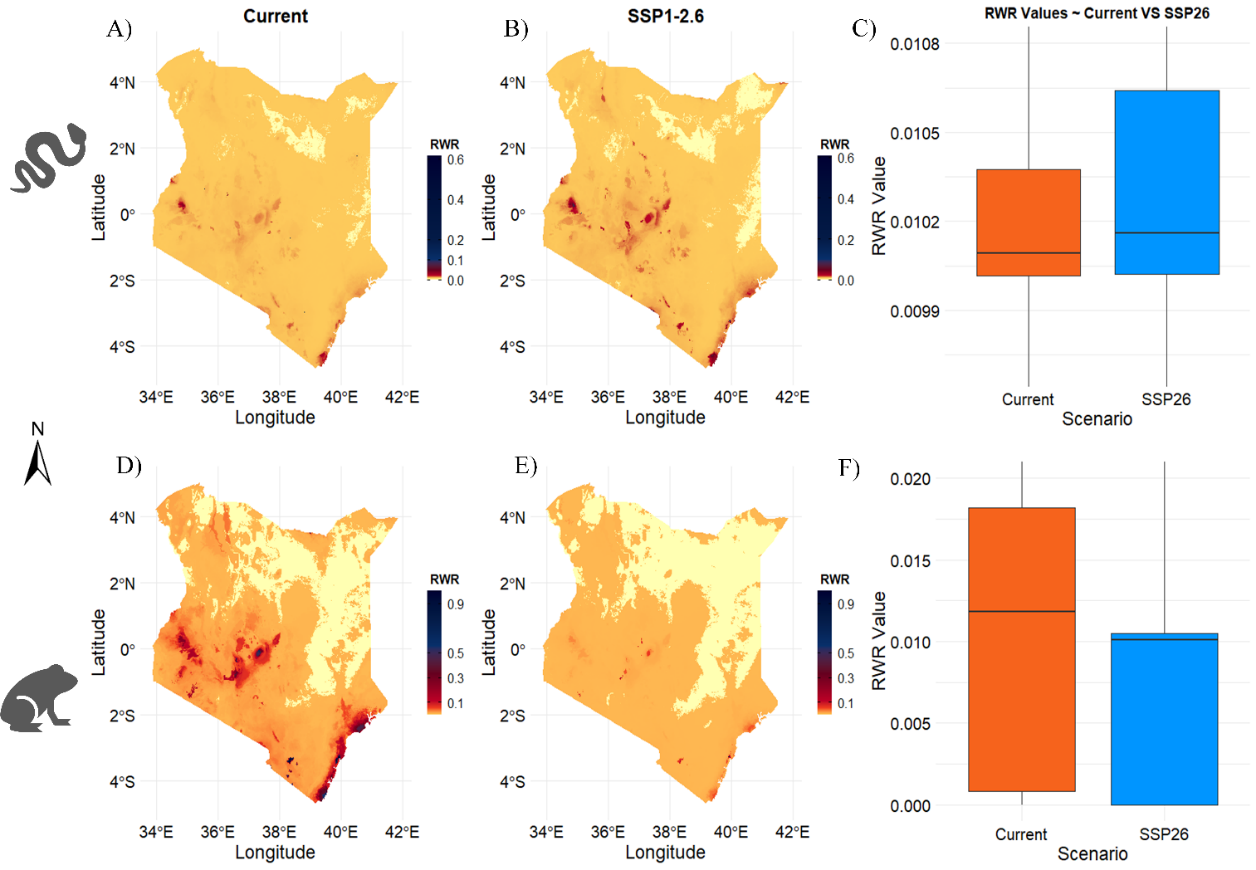


**Figure S3: RWR for reptiles and amphibians under current and future SSP1-2.6 climatic scenarios by 2050.**

**NB:** The color scale indicates lower values in yellow and higher values in dark red. C) and F); Box plots illustrating the difference between current and SSP1-2.6 RWR values. (Wilcoxon test; Z = -103, p-value = <2.2 x 10^-16^ and, Z = 416, p-value = <2.2 x 10^-16^) for C) and F), respectively. The box plots were zoomed in during plotting because more RWR values were less than 0.02, causing the box plots to be squeezed in.


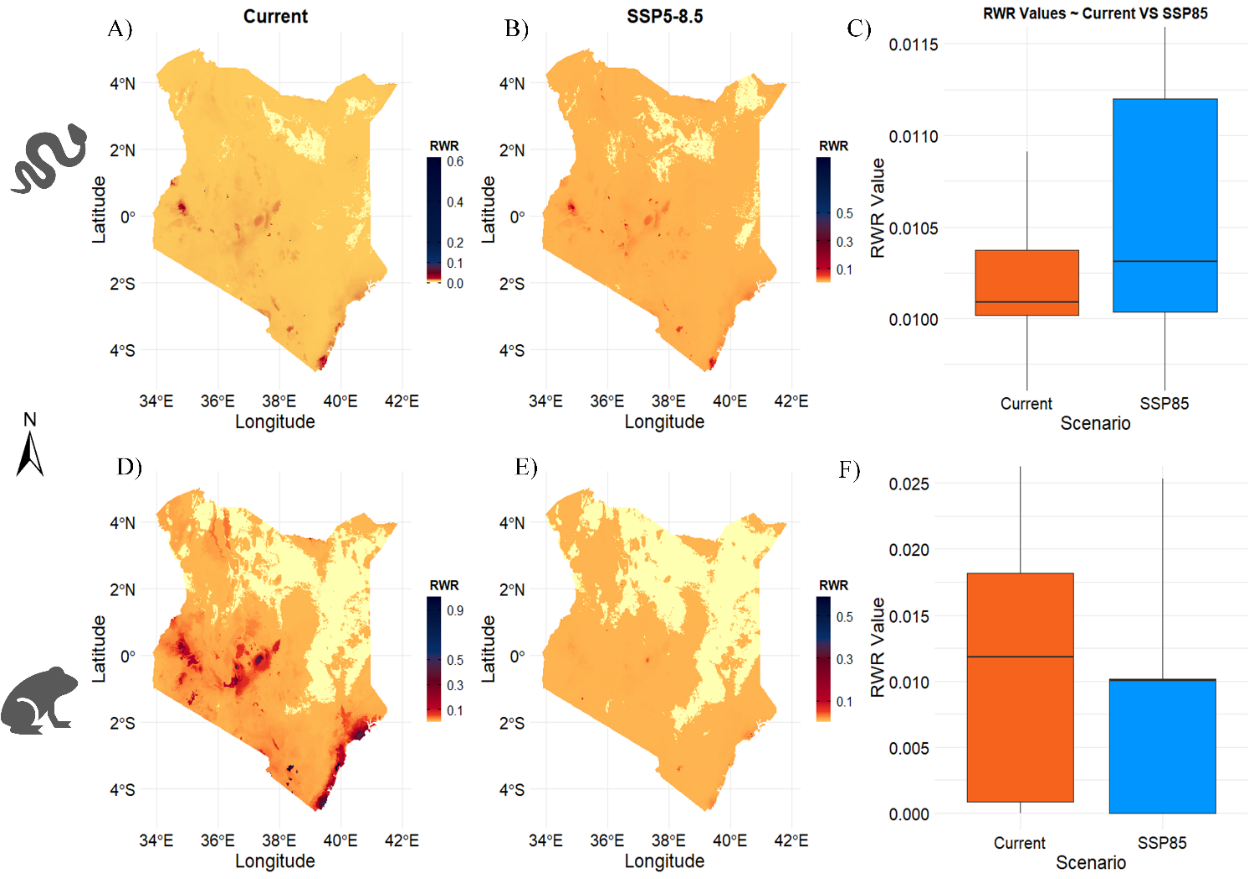


**Figure S4: RWR for reptiles and amphibians under current and future SSP5-8.5 climatic scenarios by 2050.**

**NB:** The colour scale indicates lower values in yellow and higher values in dark red. C) and F); Box plots showing the difference between current and SSP2-4.5 RWR values. (Wilcoxon test; Z = -252, p-value = <2.2 x 10^-16^ and, Z = 469, p-value = <2.2 x 10^-16^) for C) and F), respectively. The box plots were zoomed in during plotting because more RWR values were less than 0.02, causing the box plots to be squeezed in.


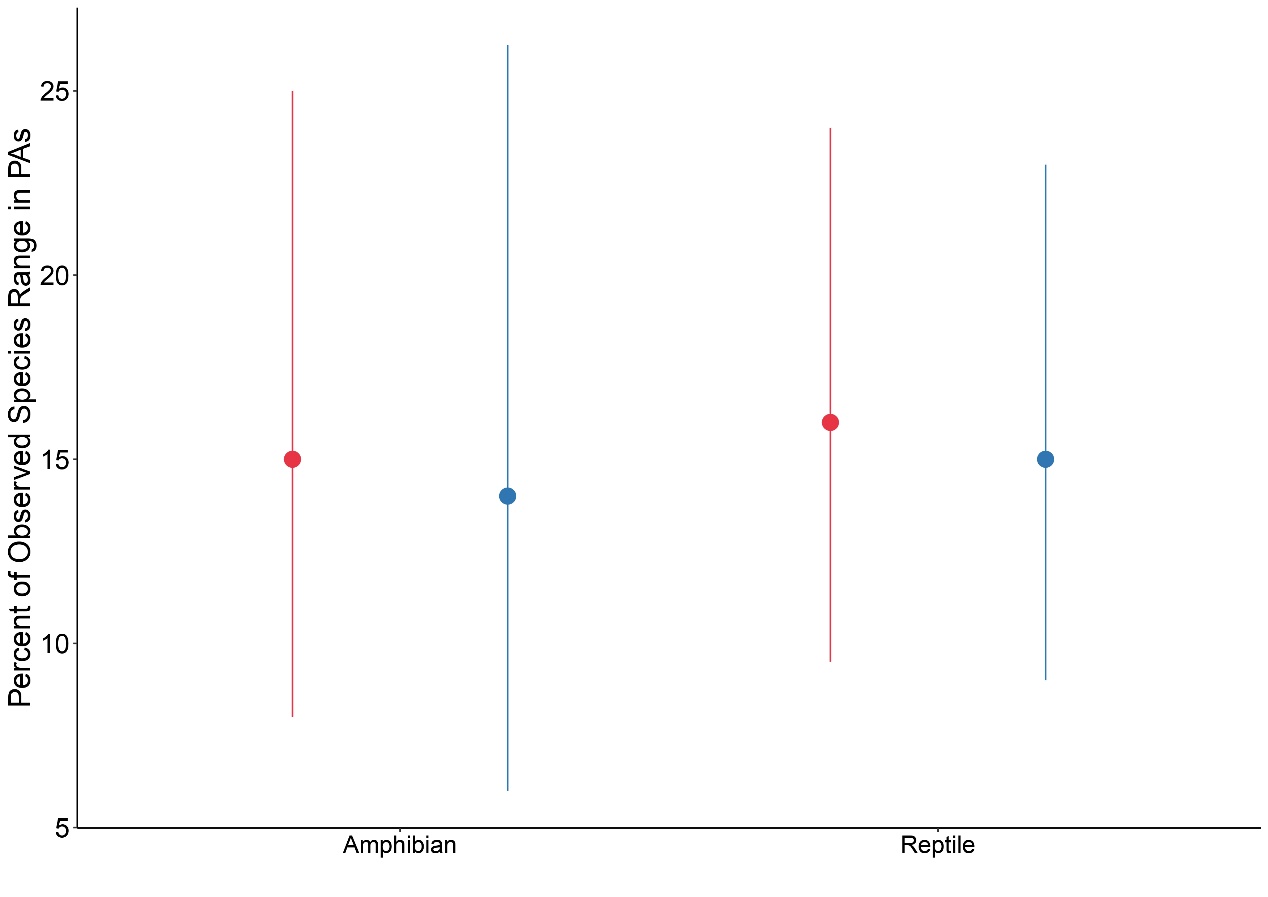


**Figure S5 Interquartile range (Q1, median, and Q3) of the percentage of species ranges distributed within protected areas. ‘Red’ is for the current scenario, and ‘blue’ is for the SSP26 scenario by 2050**


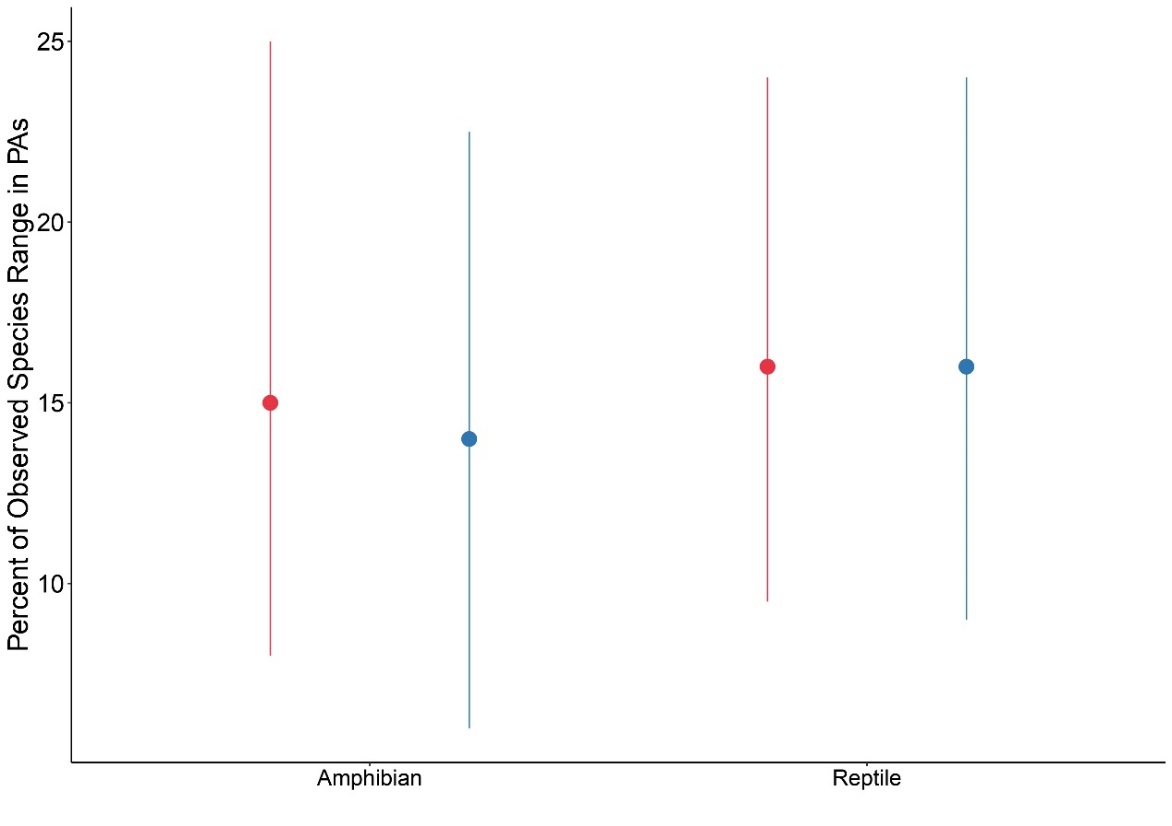


**Figure S6 Quantile range (q1, median, and q3) of the percentage of species range distributed inside protected areas. ‘Red’ is for the current scenario, and ‘blue’ is for the SSP5-8.5 scenario by 2050**


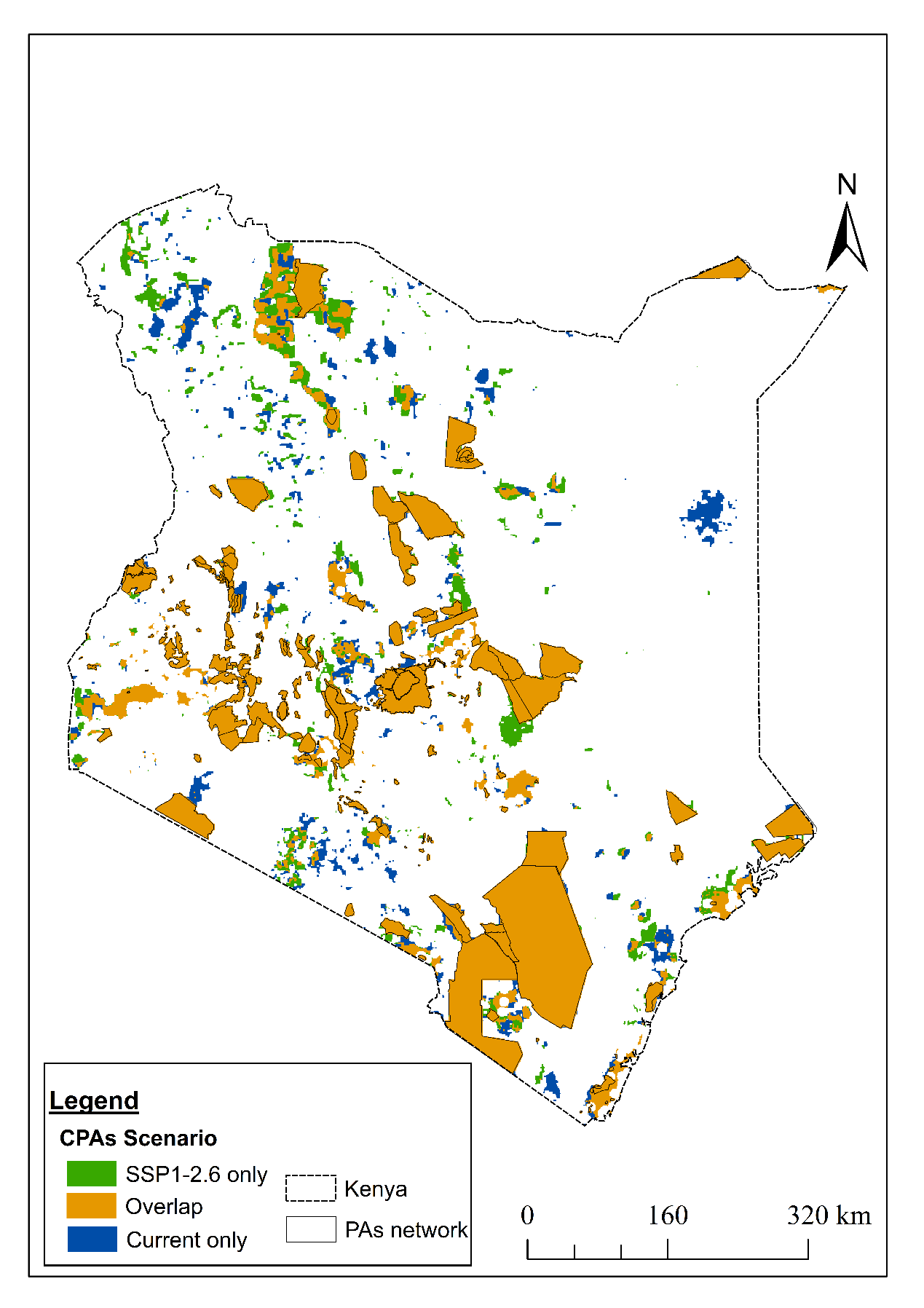


**Figure S7 Conservation Priority Areas (CPA) under SSP1- 2.6 scenario**


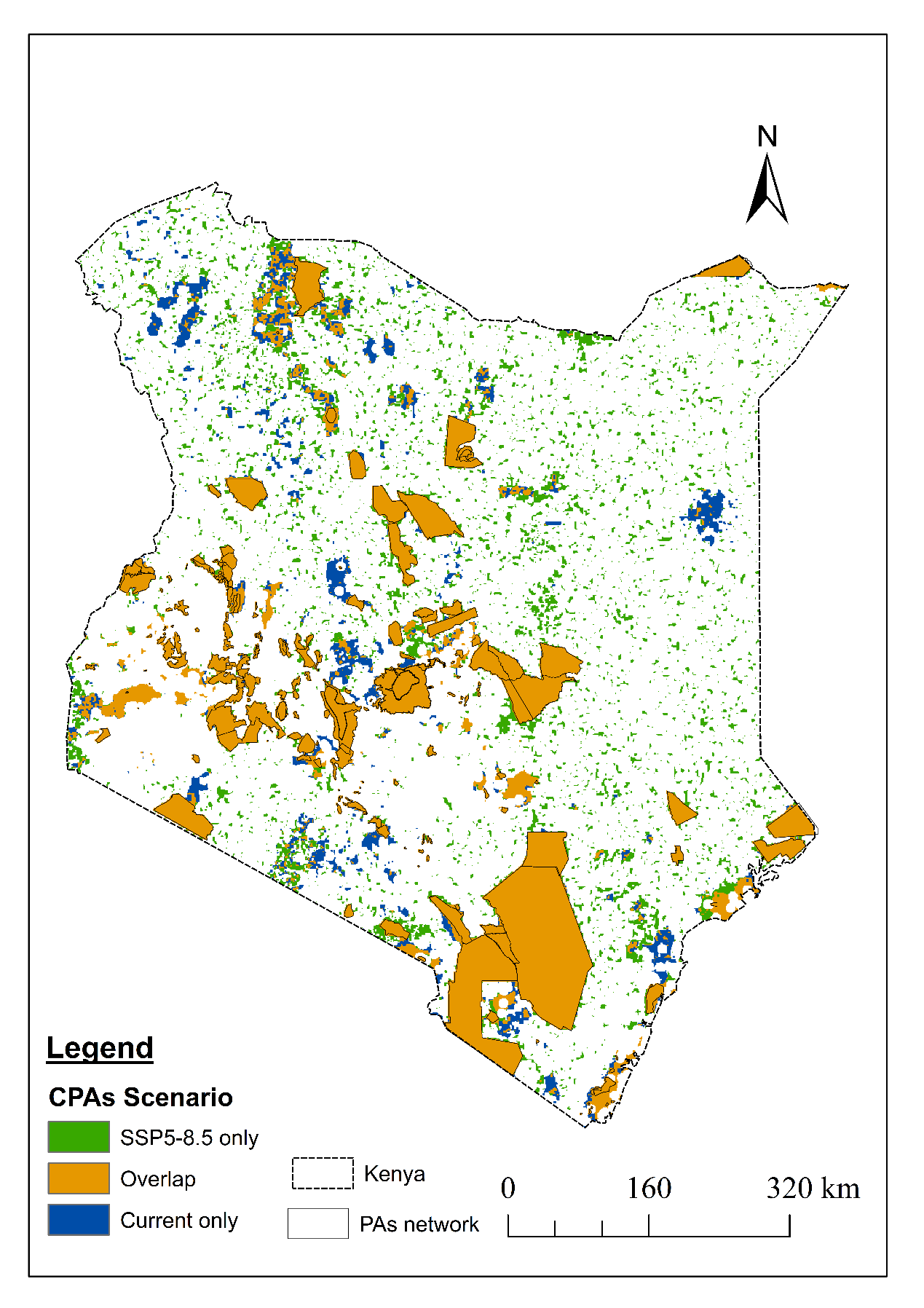


**Figure S8 Conservation Priority Areas (CPAs) under SSP5- 8.5 scenario**


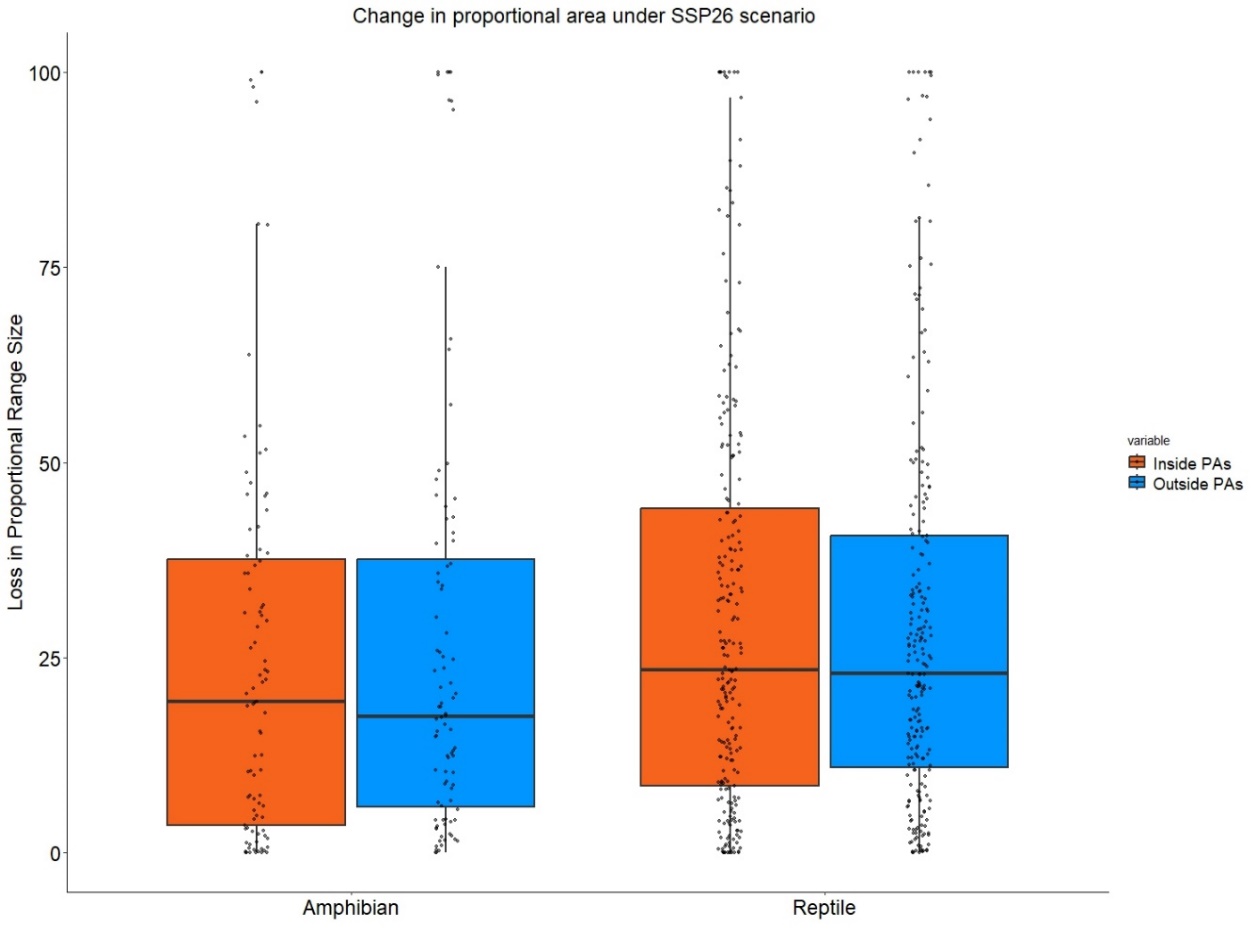


**Figure S9 Loss in proportional Range inside and outside Protected Areas (PAs) under SSP1- 2.6 scenario by 2050.**

**NB:** There was no significant difference between loss of proportional range size between inside and outside PAs (Wilcox test; p-value 0.8561 and a Z score of -0.454 for amphibians, p-value 0.8561 and a Z score of 0.182 for reptiles)


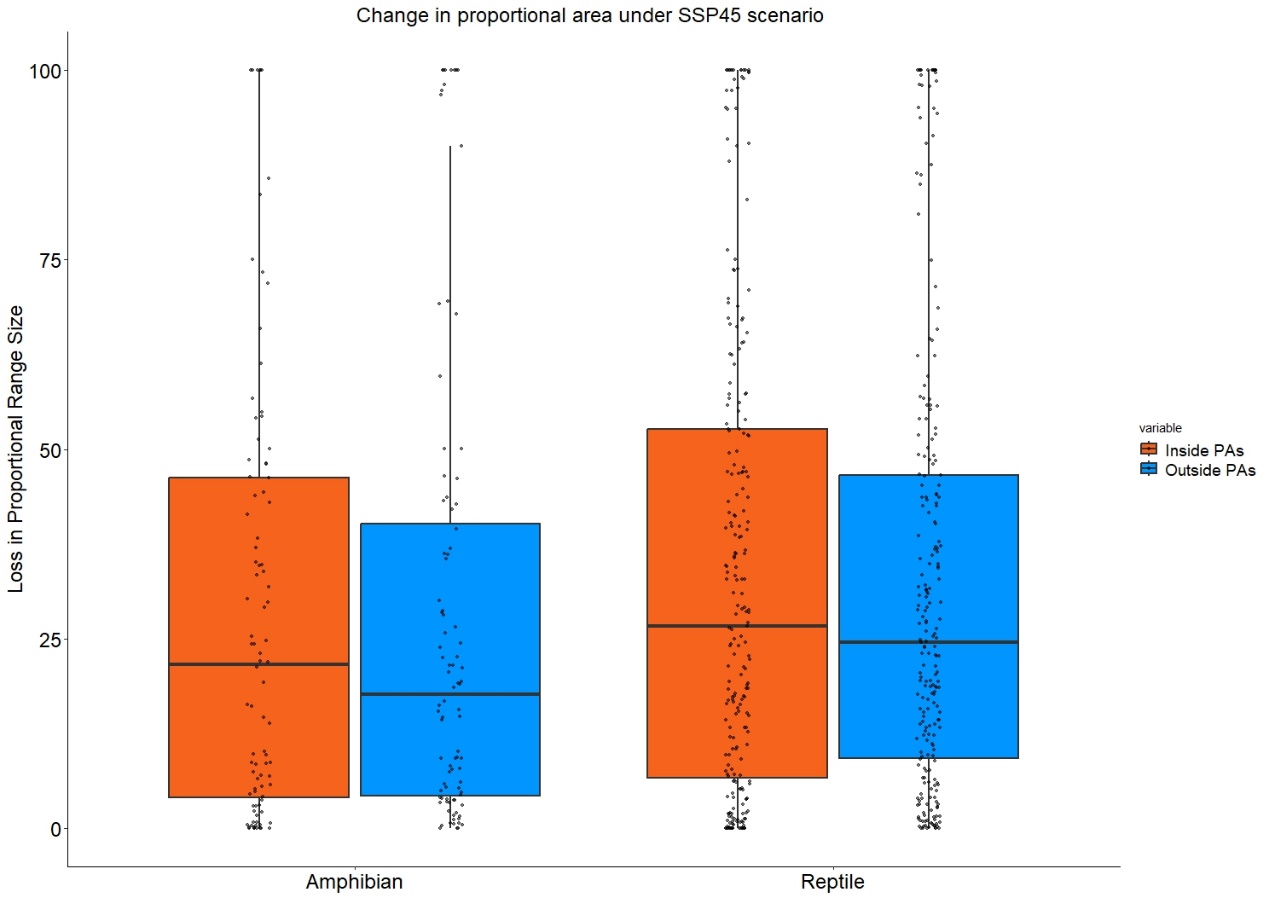


**Figure S10 Loss in Proportional Range inside and outside Protected Areas (PAs) under the SSP2-4.5 scenario by 2050.**

**NB:** There was no significant difference between the loss of proportional range size between inside and outside PAs (Wilcox test; p-value 0.9349 and a Z score of 0.0831 for amphibians; p-value 0.7817 and a Z score of 0.277 for reptiles)


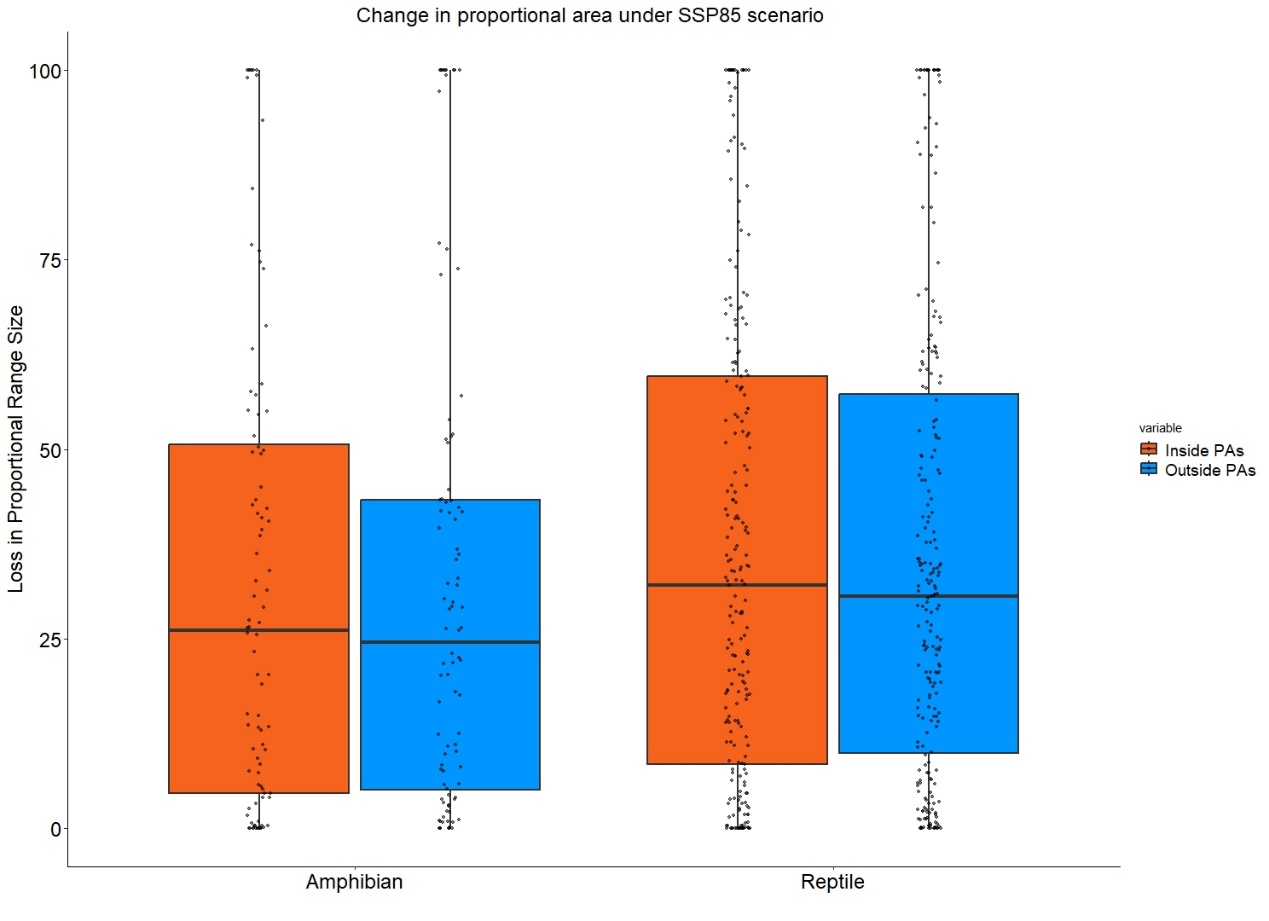


**Figure S11 Loss in Range inside and outside Protected Areas (PAs) under the SSP5-8.5 scenario by 2050.**

**NB:** There was no significant difference between loss of range size between inside and outside PAs (Wilcox test; p-value 0.9591 and a Z score of -0.0526 for amphibians, p-value 0.9585 and a Z value of 0.0523 for reptiles)


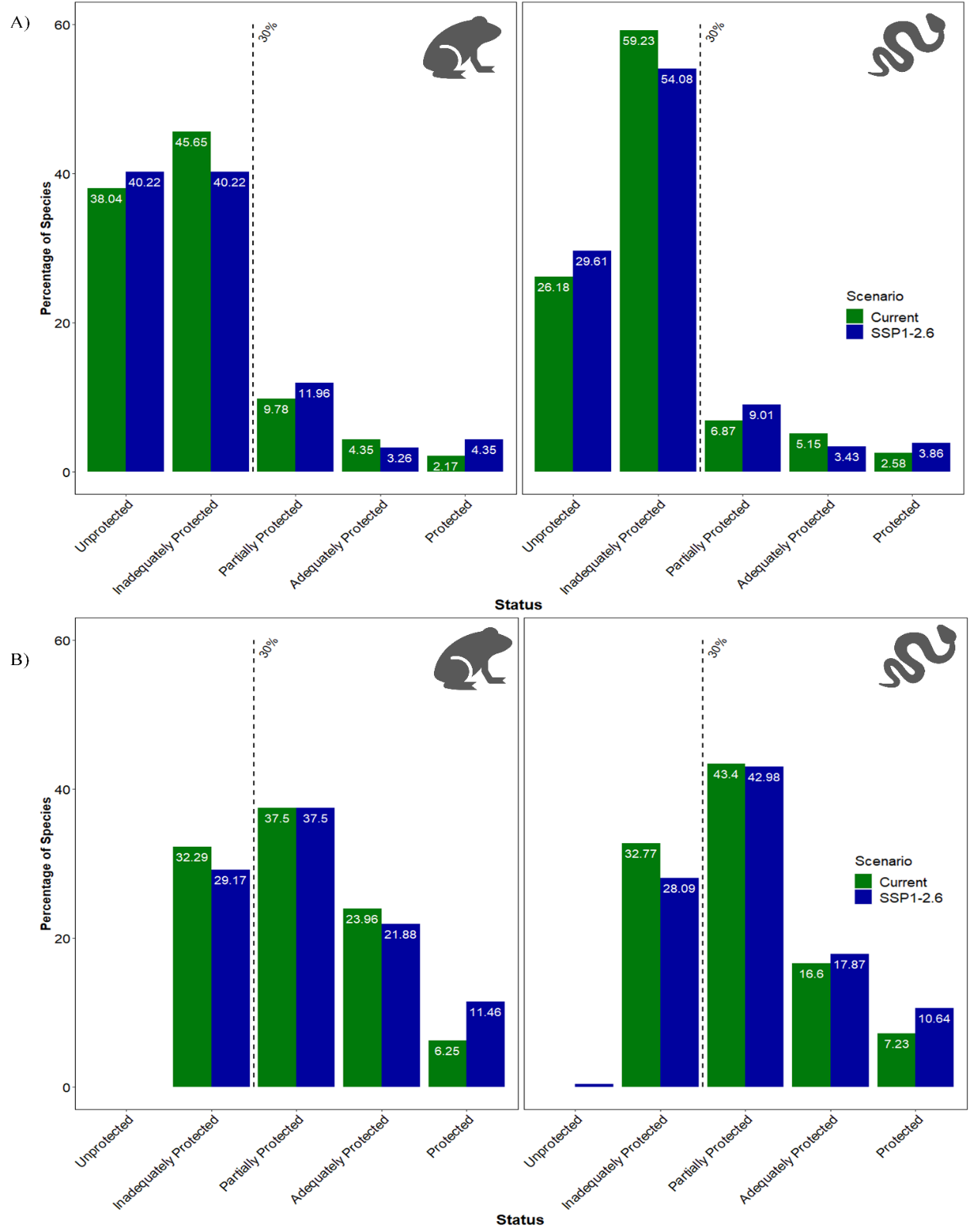


**Figure S12 Percentages of species range size inside PAs for the different conservation criteria under the SSP1-2.6 scenario by 2050**


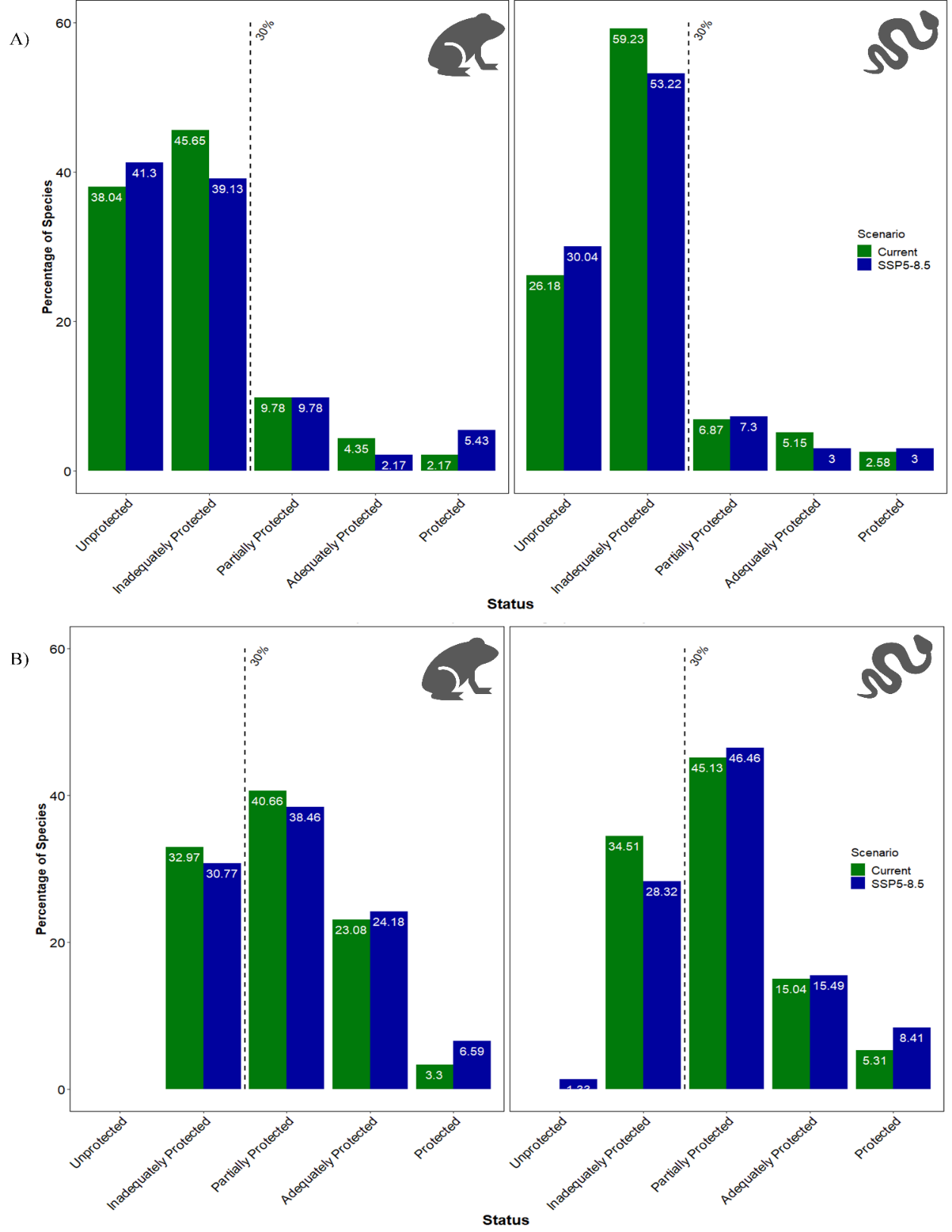


**Figure S13 Percentages of species range size inside PAs for the different conservation criteria under the SSP5-8.5 scenario by 2050**

**Table S1 Retained Bioclimatic variables and their mean and Standard Deviation (SD) of their Importance**

| **Variable** | **Mean_corTest** | **SD_corTest** |
| --- | --- | --- |
| bio_2 | 0.261831879 | 0.139868756 |
| bio_4 | 0.227224871 | 0.126499285 |
| bio_7 | 0.216919168 | 0.07307076 |
| bio_9 | 0.244403425 | 0.160809736 |
| bio_12 | 0.228950221 | 0.08015514 |
| bio_13 | 0.238590602 | 0.094508147 |
| bio_14 | 0.192381607 | 0.091432069 |
| bio_15 | 0.189442496 | 0.080786626 |
| bio_18 | 0.202176284 | 0.111210658 |
| bio_19 | 0.212660485 | 0.131061035 |

**Table S2 Species that will be locally extinct under different climatic scenarios by 2050 in Kenya**

| **Species** | **Taxonomic Group** | **Endemic** | **IUCN Category** | **Scenario** |
| --- | --- | --- | --- | --- |
| *Agama rueppelli* | Reptiles | No | LC | SSP1-2.6 |
| *Ancylodactylus laikipiensis* | Reptiles | Yes | VU | SSP1-2.6 |
| *Holodactylus africanus* | Reptiles | No | LC | SSP1-2.6 |
| *Psammophis pulcher* | Reptiles | No | LC | SSP1-2.6 |
| *Stenodactylus sthenodactylus* | Reptiles | No | LC | SSP1-2.6 |
| *Varanus exanthematicus* | Reptiles | No | LC | SSP1-2.6 |
| *Chiromantis kelleri* | Amphibians | No | LC | SSP1-2.6 |
| *Adolfus masavaensis* | Reptiles | Yes | NT | SSP2-4.5 |
| *Ancylodactylus laikipiensis* | Reptiles | Yes | VU | SSP2-4.5 |
| *Agama rueppelli* | Reptiles | No | LC | SSP2-4.5 |
| *Hemidactylus funaiolii* | Reptiles | No | DD | SSP2-4.5 |
| *Holodactylus africanus* | Reptiles | No | LC | SSP2-4.5 |
| *Psammophis pulcher* | Reptiles | No | LC | SSP2-4.5 |
| *Stenodactylus sthenodactylus* | Reptiles | No | LC | SSP2-4.5 |
| *Varanus albigularis* | Reptiles | No | LC | SSP2-4.5 |
| *Chiromantis kelleri* | Amphibians | No | LC | SSP2-4.5 |
| *Schismaderma carens* | Amphibians | No | LC | SSP2-4.5 |
| *Tomopterna gallmanni* | Amphibians | Yes | LC | SSP2-4.5 |
| *Agama rueppelli* | Reptiles | No | LC | SSP5-8.5 |
| *Ancylodactylus laikipiensis* | Reptiles | No | VU | SSP5-8.5 |
| *Ancylodactylus mathewsensis* | Reptiles | Yes | VU | SSP5-8.5 |
| *Chamaeleo laevigatus* | Reptiles | No | LC | SSP5-8.5 |
| *Cordylus tropidosternum* | Reptiles | No | LC | SSP5-8.5 |
| *Crotaphopeltis tornieri* | Reptiles | No | LC | SSP5-8.5 |
| *Cryptoblepharus africanus* | Reptiles | No | LC | SSP5-8.5 |
| *Hemidactylus funaiolii* | Reptiles | No | DD | SSP5-8.5 |
| *Holodactylus africanus* | Reptiles | No | LC | SSP5-8.5 |
| *Lygodactylus scheffleri* | Reptiles | No | DD | SSP5-8.5 |
| *Psammophis pulcher* | Reptiles | No | LC | SSP5-8.5 |
| *Stenodactylus sthenodactylus* | Reptiles | No | LC | SSP5-8.5 |
| *Trachylepis dichroma* | Reptiles | No | LC | SSP5-8.5 |
| *Varanus albigularis* | Reptiles | No | LC | SSP5-8.5 |
| *Chiromantis kelleri* | Amphibians | No | LC | SSP5-8.5 |
| *Leptopelis flavomaculatus* | Amphibians | No | LC | SSP5-8.5 |
| *Amietia wittei* | Amphibians | No | LC | SSP5-8.5 |
| *Tomopterna gallmanni* | Amphibians | Yes | LC | SSP5-8.5 |

**Note:** The abbreviations in IUCN category stands for LC; Least Concern, VU; Vulnerable, NT; Near Threatened, DD; Data Deficient, EN; Endangered, N/A; Recently described whose status hasn’t been assessed yet

**Table S3 Species that have their range size entirely outside the existing Protected Areas (PAs) network of Kenya in different scenarios**

| **Species** | **Taxonomic Group** | **Endemic** | **IUCN Category** | **Scenario** |
| --- | --- | --- | --- | --- |
| *Ancylodactylus laikipiensis* | Reptiles | Yes | VU | Current |
| *Grayia smithii* | Reptiles | No | LC | Current |
| *Pelusios williamsi* | Reptiles | No | LC | Current |
| *Philochortus rudolfensis* | Reptiles | No | DD | Current |
| *Psammophis pulcher* | Reptiles | No | LC | Current |
| *Boulengerula niedeni* | Amphibians | Yes | EN | Current |
| *Callulina kreffti* | Amphibians | No | LC | Current |
| *Hemisus guineensis* | Amphibians | No | LC | Current |
| *Hyperolius substriatus* | Amphibians | No | LC | Current |
| *Chamaeleo laevigatus* | Reptiles | No | LC | SSP1-2.6 |
| *Grayia smithii* | Reptiles | No | LC | SSP1-2.6 |
| *Letheobia mbeerensis* | Reptiles | Yes | N/A | SSP1-2.6 |
| *Pelusios williamsi* | Reptiles | No | LC | SSP1-2.6 |
| *Philochortus rudolfensis* | Reptiles | No | DD | SSP1-2.6 |
| *Boulengerula niedeni* | Amphibians | Yes | EN | SSP1-2.6 |
| *Callulina kreffti* | Amphibians | No | LC | SSP1-2.6 |
| *Hemisus guineensis* | Amphibians | No | LC | SSP1-2.6 |
| *Hyperolius substriatus* | Amphibians | No | LC | SSP1-2.6 |
| *Tomopterna gallmanni* | Amphibians | Yes | LC | SSP1-2.6 |
| *Boulengerula niedeni* | Amphibians | Yes | EN | SSP2-4.5 |
| *Callulina kreffti* | Amphibians | No | LC | SSP2-4.5 |
| *Hemisus guineensis* | Amphibians | No | LC | SSP2-4.5 |
| *Hyperolius substriatus* | Amphibians | No | LC | SSP2-4.5 |
| *Crotaphopeltis tornieri* | Reptiles | No | LC | SSP2-4.5 |
| *Grayia smithii* | Reptiles | No | LC | SSP2-4.5 |
| *Letheobia mbeerensis* | Reptiles | Yes | N/A | SSP2-4.5 |
| *Lygodactylus scheffleri* | Reptiles | No | DD | SSP2-4.5 |
| *Pelusios williamsi* | Reptiles | No | LC | SSP2-4.5 |
| *Philochortus rudolfensis* | Reptiles | No | DD | SSP2-4.5 |
| *Psammophis pulcher* | Reptiles | No | LC | SSP2-4.5 |

**Note:** The abbreviations in IUCN category stands for LC; Least Concern, VU; Vulnerable, NT; Near Threatened, DD; Data Deficient, EN; Endangered, N/A; Recently described whose status hasn’t been assessed yet

**Table S3 Species that have their range size entirely outside the existing Protected Areas (PAs) network of Kenya in different scenarios (Continued)**

| **Species** | **Taxonomic Group** | **Endemic** | **IUCN Category** | **Scenario** |
| --- | --- | --- | --- | --- |
| *Grayia smithii* | Reptiles | No | LC | SSP5-8.5 |
| *Leptotyphlops keniensis* | Reptiles | Yes | DD | SSP5-8.5 |
| *Letheobia mbeerensis* | Reptiles | Yes | N/A | SSP5-8.5 |
| *Pelusios williamsi* | Reptiles | No | LC | SSP5-8.5 |
| *Philochortus rudolfensis* | Reptiles | No | DD | SSP5-8.5 |
| *Pseuderemias smithii* | Reptiles | No | LC | SSP5-8.5 |
| *Boulengerula niedeni* | Amphibians | Yes | EN | SSP5-8.5 |
| *Callulina kreffti* | Amphibians | No | LC | SSP5-8.5 |
| *Hemisus guineensis* | Amphibians | No | LC | SSP5-8.5 |
| *Hyperolius substriatus* | Amphibians | No | LC | SSP5-8.5 |

**Note:** The abbreviations in IUCN category stands for LC; Least Concern, VU; Vulnerable, NT; Near Threatened, DD; Data Deficient, EN; Endangered, N/A; Recently described whose status hasn’t been assessed yet

**Table S4 Area coverage of the existing Protected Areas (PAs) and Conservation Protected Areas (CPAs)**

| **Scenario** | **Area (km2)** | **% of Kenya`s Total Area** |
| --- | --- | --- |
| Existing Protected Areas (PAs) | 62,247 | 10.6 |
| Current CPAs (based on current species distribution) | 95,988 | 16.4 |
| SSP2-4.5 CPAs (SSP2-4.5 Scenario) | 95,151 | 16.2 |
| SSP1-2.6 CPAs (SSP1-2.6 Scenario) | 95,899 | 16.4 |
| SSP5-8.5 CPAs (SSP5-8.5 Scenario) | 116,445 | 19.9 |

**Table S5 Ensemble Model performance of each species studied**

| **Species** | **Taxonomic Group** | **AUC** | **TSS** | **Threshold** |
| --- | --- | --- | --- | --- |
| *Afrixalus delicatus* | Amphibian | 0.9703 | 0.9421 | 0.2170 |
| *Afrixalus fornasini* | Amphibian | 0.9729 | 0.9349 | 0.1100 |
| *Afrixalus fulvovittatus* | Amphibian | 0.9469 | 0.8675 | 0.0919 |
| *Afrixalus osorioi* | Amphibian | 0.9970 | 0.9946 | 0.2836 |
| *Afrixalus septentrionalis* | Amphibian | 0.9387 | 0.8314 | 0.1578 |
| *Afrixalus sylvaticus* | Amphibian | 0.9989 | 0.9970 | 0.6153 |
| *Amietia nutti* | Amphibian | 0.9453 | 0.8619 | 0.1306 |
| *Amietia wittei* | Amphibian | 0.9874 | 0.9752 | 0.2475 |
| *Arthroleptis stenodactylus* | Amphibian | 0.9624 | 0.9362 | 0.3285 |
| *Boulengerula changamwensis* | Amphibian | 0.9857 | 0.9765 | 0.3844 |
| *Boulengerula denhardti* | Amphibian | 0.9934 | 0.9897 | 0.3537 |
| *Boulengerula niedeni* | Amphibian | 0.9905 | 0.9806 | 0.4948 |
| *Boulengerula taitana* | Amphibian | 0.9721 | 0.9412 | 0.4264 |
| *Bufo garmani* | Amphibian | 0.9239 | 0.8113 | 0.1081 |
| *Bufo gutturalis* | Amphibian | 0.9008 | 0.7750 | 0.1105 |
| *Bufo kerinyagae* | Amphibian | 0.9986 | 0.9978 | 0.6429 |
| *Bufo lonnbergi* | Amphibian | 0.9868 | 0.9859 | 0.4731 |
| *Bufo lughensis* | Amphibian | 0.9029 | 0.8169 | 0.2758 |
| *Bufo maculatus* | Amphibian | 0.9713 | 0.9257 | 0.2586 |
| *Bufo regularis* | Amphibian | 0.9371 | 0.8194 | 0.1954 |
| *Bufo steindachneri* | Amphibian | 0.9906 | 0.9784 | 0.2557 |
| *Bufo xeros* | Amphibian | 0.9207 | 0.8317 | 0.1072 |
| *Cacosternum boettgeri* | Amphibian | 0.9911 | 0.9742 | 0.3408 |
| *Callulina kreffti* | Amphibian | 0.9992 | 0.9976 | 0.5841 |
| *Chiromantis kelleri* | Amphibian | 0.9696 | 0.9510 | 0.3460 |
| *Chiromantis petersii* | Amphibian | 0.9110 | 0.7630 | 0.1644 |
| *Chiromantis xerampelina* | Amphibian | 0.9589 | 0.8610 | 0.0902 |
| *Hemisus guineensis* | Amphibian | 0.9932 | 0.9902 | 0.3378 |
| *Hemisus marmoratus* | Amphibian | 0.9412 | 0.8061 | 0.1698 |
| *Hoplobatrachus occipitalis* | Amphibian | 0.9277 | 0.8185 | 0.1595 |
| *Hyperolius argus* | Amphibian | 0.9906 | 0.9775 | 0.2479 |

**Table S5 Ensemble Model performance of each species studied (continued)**

| **Species** | **Taxonomic Group** | **AUC** | **TSS** | **Threshold** |
| --- | --- | --- | --- | --- |
| *Hyperolius cinnamomeoventris* | Amphibian | 0.9730 | 0.9218 | 0.1157 |
| *Hyperolius cystocandicans* | Amphibian | 0.9824 | 0.9532 | 0.2040 |
| *Hyperolius glandicolor* | Amphibian | 0.9281 | 0.8044 | 0.0775 |
| *Hyperolius howelli* | Amphibian | 0.9690 | 0.9285 | 0.2603 |
| *Hyperolius kivuensis* | Amphibian | 0.9476 | 0.8663 | 0.1435 |
| *Hyperolius lateralis* | Amphibian | 0.9847 | 0.9665 | 0.2500 |
| *Hyperolius mariae* | Amphibian | 0.9881 | 0.9867 | 0.3215 |
| *Hyperolius microps* | Amphibian | 0.9794 | 0.9600 | 0.3767 |
| *Hyperolius montanus* | Amphibian | 0.9678 | 0.8900 | 0.1445 |
| *Hyperolius nasutus* | Amphibian | 0.9775 | 0.9516 | 0.2198 |
| *Hyperolius parkeri* | Amphibian | 0.9964 | 0.9896 | 0.3506 |
| *Hyperolius pusillus* | Amphibian | 0.9603 | 0.8355 | 0.1581 |
| *Hyperolius rubrovermiculatus* | Amphibian | 0.9988 | 0.9947 | 0.5525 |
| *Hyperolius substriatus* | Amphibian | 0.9675 | 0.9480 | 0.4091 |
| *Hyperolius tuberilinguis* | Amphibian | 0.9837 | 0.9431 | 0.1419 |
| *Hyperolius viridiflavus* | Amphibian | 0.9213 | 0.7668 | 0.2067 |
| *Kassina maculata* | Amphibian | 0.9821 | 0.9611 | 0.4003 |
| *Kassina senegalensis* | Amphibian | 0.9157 | 0.7619 | 0.1225 |
| *Kassina somalica* | Amphibian | 0.8976 | 0.8479 | 0.1605 |
| *Leptopelis argenteus* | Amphibian | 0.9949 | 0.9888 | 0.3174 |
| *Leptopelis bocagii* | Amphibian | 0.9631 | 0.9427 | 0.2508 |
| *Leptopelis flavomaculatus* | Amphibian | 0.9906 | 0.9843 | 0.3774 |
| *Mertensophryne lonnbergi* | Amphibian | 0.9881 | 0.9754 | 0.2753 |
| *Mertensophryne micranotis* | Amphibian | 0.9803 | 0.9484 | 0.1670 |
| *Mertensophryne taitana* | Amphibian | 0.9602 | 0.8608 | 0.0664 |
| *Phrynobatrachus acridoides* | Amphibian | 0.9165 | 0.7749 | 0.1528 |
| *Phrynobatrachus graueri* | Amphibian | 0.9985 | 0.9973 | 0.5517 |
| *Phrynobatrachus keniensis* | Amphibian | 0.9740 | 0.9466 | 0.2321 |
| *Phrynobatrachus kinangopensis* | Amphibian | 0.9677 | 0.9328 | 0.1194 |
| *Phrynobatrachus mababiensis* | Amphibian | 0.9530 | 0.9113 | 0.2536 |
| *Phrynobatrachus natalensis* | Amphibian | 0.9143 | 0.7928 | 0.2064 |
| *Phrynobatrachus scheffleri* | Amphibian | 0.9434 | 0.9104 | 0.2262 |

**Table S5 Ensemble Model performance of each species studied (continued)**

| **Species** | **Taxonomic Group** | **AUC** | **TSS** | **Threshold** |
| --- | --- | --- | --- | --- |
| *Phrynobatrachus ukingensis* | Amphibian | 0.9356 | 0.9166 | 0.5399 |
| *Phrynomantis bifasciatus* | Amphibian | 0.9236 | 0.7676 | 0.0834 |
| *Phrynomantis somalicus* | Amphibian | 0.9739 | 0.9271 | 0.1829 |
| *Poyntonophrynus lughensis* | Amphibian | 0.9237 | 0.8030 | 0.1536 |
| *Ptychadena anchietae* | Amphibian | 0.9130 | 0.7270 | 0.1911 |
| *Ptychadena mahnerti* | Amphibian | 0.9846 | 0.9776 | 0.4466 |
| *Ptychadena mascareniensis* | Amphibian | 0.9401 | 0.8070 | 0.1497 |
| *Ptychadena mossambica* | Amphibian | 0.9692 | 0.9397 | 0.3382 |
| *Ptychadena nilotica* | Amphibian | 0.9616 | 0.9229 | 0.1895 |
| *Ptychadena oxyrhynchus* | Amphibian | 0.9516 | 0.9208 | 0.1794 |
| *Ptychadena schillukorum* | Amphibian | 0.9306 | 0.8589 | 0.2314 |
| *Ptychadena superciliaris* | Amphibian | 0.9085 | 0.8321 | 0.0963 |
| *Pyxicephalus edulis* | Amphibian | 0.8797 | 0.7578 | 0.0412 |
| *Rana angolensis* | Amphibian | 0.9392 | 0.8388 | 0.1291 |
| *Rana wittei* | Amphibian | 0.9952 | 0.9930 | 0.4142 |
| *Schismaderma carens* | Amphibian | 0.9875 | 0.9710 | 0.4044 |
| *Schistometopum gregorii* | Amphibian | 0.9799 | 0.9642 | 0.1834 |
| *Sclerophrys garmani* | Amphibian | 0.9325 | 0.7575 | 0.0846 |
| *Sclerophrys gutturalis* | Amphibian | 0.9205 | 0.7588 | 0.1525 |
| *Sclerophrys kerinyagae* | Amphibian | 0.9688 | 0.8981 | 0.1926 |
| *Sclerophrys kisoloensis* | Amphibian | 0.9529 | 0.9060 | 0.4465 |
| *Sclerophrys pusilla* | Amphibian | 0.9363 | 0.8031 | 0.1587 |
| *Sclerophrys regularis* | Amphibian | 0.9577 | 0.8719 | 0.1448 |
| *Sclerophrys steindachneri* | Amphibian | 0.9452 | 0.8828 | 0.2480 |
| *Sclerophrys turkanae* | Amphibian | 0.9549 | 0.8983 | 0.1640 |
| *Sclerophrys xeros* | Amphibian | 0.9312 | 0.7825 | 0.1616 |
| *Tomopterna cryptotis* | Amphibian | 0.9118 | 0.7614 | 0.0373 |
| *Tomopterna gallmanni* | Amphibian | 0.9889 | 0.9794 | 0.3663 |
| *Tomopterna marmorata* | Amphibian | 0.9271 | 0.8564 | 0.1653 |
| *Tomopterna wambensis* | Amphibian | 0.9316 | 0.8970 | 0.2290 |
| *Xenopus borealis* | Amphibian | 0.9259 | 0.7739 | 0.1445 |
| *Xenopus laevis* | Amphibian | 0.9230 | 0.8749 | 0.1189 |

**Table S5 Ensemble Model performance of each species studied (continued)**

| **Species** | **Taxonomic Group** | **AUC** | **TSS** | **Threshold** |
| --- | --- | --- | --- | --- |
| *Xenopus muelleri* | Amphibian | 0.9663 | 0.9066 | 0.1372 |
| *Xenopus victorianus* | Amphibian | 0.9441 | 0.8469 | 0.1337 |
| *Acanthocercus gregorii* | Reptile | 0.9522 | 0.8619 | 0.1755 |
| *Acanthocercus minutus* | Reptile | 0.9707 | 0.9492 | 0.3110 |
| *Acanthocercus ugandaensis* | Reptile | 0.9651 | 0.9062 | 0.0645 |
| *Acontias percivali* | Reptile | 0.9897 | 0.9888 | 0.3625 |
| *Adolfus alleni* | Reptile | 0.9924 | 0.9861 | 0.3121 |
| *Adolfus jacksoni* | Reptile | 0.9505 | 0.8688 | 0.1139 |
| *Adolfus kibonotensis* | Reptile | 0.9652 | 0.8800 | 0.0981 |
| *Adolfus masavaensis* | Reptile | 0.9980 | 0.9960 | 0.4279 |
| *Afrotyphlops angolensis* | Reptile | 0.9623 | 0.9180 | 0.2111 |
| *Afrotyphlops brevis* | Reptile | 0.9475 | 0.8780 | 0.1525 |
| *Afrotyphlops lineolatus* | Reptile | 0.9351 | 0.8001 | 0.1660 |
| *Afrotyphlops mucruso* | Reptile | 0.9847 | 0.9519 | 0.2605 |
| *Agama agama* | Reptile | 0.9183 | 0.8030 | 0.1406 |
| *Agama armata* | Reptile | 0.9335 | 0.8866 | 0.2105 |
| *Agama caudospinosa* | Reptile | 0.9444 | 0.8551 | 0.1451 |
| *Agama hulbertorum* | Reptile | 0.9753 | 0.9527 | 0.2764 |
| *Agama kaimosae* | Reptile | 0.9696 | 0.9560 | 0.2022 |
| *Agama lionotus* | Reptile | 0.9486 | 0.7858 | 0.3379 |
| *Agama mwanzae* | Reptile | 0.9943 | 0.9730 | 0.2959 |
| *Agama persimilis* | Reptile | 0.9226 | 0.7773 | 0.0439 |
| *Agama rueppelli* | Reptile | 0.7780 | 0.7380 | 0.3011 |
| *Amblyodipsas polylepis* | Reptile | 0.9906 | 0.9746 | 0.2661 |
| *Amblyodipsas unicolor* | Reptile | 0.9576 | 0.9365 | 0.1498 |
| *Ancylodactylus africanus* | Reptile | 0.9648 | 0.9393 | 0.3857 |
| *Ancylodactylus elgonensis* | Reptile | 0.9849 | 0.9763 | 0.3437 |
| *Ancylodactylus kenyaensis* | Reptile | 0.9795 | 0.9613 | 0.5268 |
| *Ancylodactylus kituiensis* | Reptile | 0.9829 | 0.9669 | 0.4427 |
| *Ancylodactylus laikipiensis* | Reptile | 0.9980 | 0.9973 | 0.5886 |
| *Ancylodactylus mathewsensis* | Reptile | 0.9914 | 0.9849 | 0.3136 |
| *Ancylodactylus spawlsi* | Reptile | 0.9787 | 0.9499 | 0.3454 |

**Table S5 Ensemble Model performance of each species studied (continued)**

| **Species** | **Taxonomic Group** | **AUC** | **TSS** | **Threshold** |
| --- | --- | --- | --- | --- |
| *Aparallactus capensis* | Reptile | 0.9986 | 0.9974 | 0.5733 |
| *Aparallactus guentheri* | Reptile | 0.9676 | 0.9174 | 0.2057 |
| *Aparallactus jacksonii* | Reptile | 0.9134 | 0.7650 | 0.1445 |
| *Aparallactus lunulatus* | Reptile | 0.9060 | 0.7894 | 0.0852 |
| *Aparallactus turneri* | Reptile | 0.9768 | 0.9390 | 0.3110 |
| *Atheris desaixi* | Reptile | 0.9942 | 0.9789 | 0.3138 |
| *Atheris hispida* | Reptile | 0.9944 | 0.9889 | 0.5698 |
| *Atheris squamigera* | Reptile | 0.9690 | 0.9515 | 0.3643 |
| *Atractaspis bibronii* | Reptile | 0.9442 | 0.9031 | 0.1911 |
| *Atractaspis fallax* | Reptile | 0.9053 | 0.7746 | 0.1590 |
| *Atractaspis irregularis* | Reptile | 0.9192 | 0.8123 | 0.0947 |
| *Atractaspis microlepidota* | Reptile | 0.9351 | 0.8999 | 0.1390 |
| *Bitis arietans* | Reptile | 0.9340 | 0.7885 | 0.1559 |
| *Bitis gabonica* | Reptile | 0.9710 | 0.9525 | 0.3142 |
| *Bitis nasicornis* | Reptile | 0.9414 | 0.8909 | 0.2320 |
| *Bitis worthingtoni* | Reptile | 0.9806 | 0.9414 | 0.1409 |
| *Boaedon capensis* | Reptile | 0.9313 | 0.7861 | 0.1776 |
| *Boaedon fradei* | Reptile | 0.9256 | 0.9228 | 0.2651 |
| *Boaedon fuliginosus* | Reptile | 0.9300 | 0.7993 | 0.0862 |
| *Boaedon olivaceus* | Reptile | 0.9825 | 0.9707 | 0.3184 |
| *Boaedon paralineatus* | Reptile | 0.9680 | 0.9582 | 0.3743 |
| *Broadleysaurus major* | Reptile | 0.9138 | 0.7798 | 0.1978 |
| *Causus defilippii* | Reptile | 0.9836 | 0.9695 | 0.3238 |
| *Causus lichtensteinii* | Reptile | 0.9928 | 0.9845 | 0.4335 |
| *Causus resimus* | Reptile | 0.9363 | 0.8202 | 0.1433 |
| *Causus rhombeatus* | Reptile | 0.9457 | 0.8463 | 0.2221 |
| *Chalcides bottegi* | Reptile | 0.9890 | 0.9730 | 0.2244 |
| *Chamaeleo dilepis quilensis* | Reptile | 0.9521 | 0.9234 | 0.3194 |
| *Chamaeleo dilepis roper* | Reptile | 0.9899 | 0.9772 | 0.4933 |
| *Chamaeleo gracilis* | Reptile | 0.9420 | 0.7350 | 0.1196 |
| *Chamaeleo hoehnelii* | Reptile | 0.9591 | 0.9517 | 0.2617 |
| *Chamaeleo laevigatus* | Reptile | 0.9777 | 0.9769 | 0.4377 |

**Table S5 Ensemble Model performance of each species studied (continued)**

| **Species** | **Taxonomic Group** | **AUC** | **TSS** | **Threshold** |
| --- | --- | --- | --- | --- |
| *Chamaesaura tenuior* | Reptile | 0.9595 | 0.9043 | 0.1760 |
| *Chelonia mydas* | Reptile | 0.9904 | 0.9710 | 0.1517 |
| *Chondrodactylus turneri* | Reptile | 0.9721 | 0.9499 | 0.3233 |
| *Cnemaspis dickersonae* | Reptile | 0.9810 | 0.9753 | 0.2638 |
| *Coluber florulentus* | Reptile | 0.9883 | 0.9758 | 0.5106 |
| *Cordylus tropidosternum* | Reptile | 0.9953 | 0.9887 | 0.5686 |
| *Crocodylus niloticus* | Reptile | 0.9440 | 0.8121 | 0.2469 |
| *Crotaphopeltis braestrupi* | Reptile | 0.9821 | 0.9590 | 0.1706 |
| *Crotaphopeltis degeni* | Reptile | 0.9413 | 0.8616 | 0.0830 |
| *Crotaphopeltis hotamboeia* | Reptile | 0.9063 | 0.7378 | 0.1479 |
| *Crotaphopeltis tornieri* | Reptile | 0.9939 | 0.9903 | 0.6109 |
| *Cryptoblepharus africanus* | Reptile | 0.9976 | 0.9956 | 0.4983 |
| *Dasypeltis atra* | Reptile | 0.9323 | 0.8204 | 0.0835 |
| *Dasypeltis medici* | Reptile | 0.9448 | 0.8717 | 0.1154 |
| *Dasypeltis scabra* | Reptile | 0.9230 | 0.7080 | 0.2390 |
| *Dendroaspis angusticeps* | Reptile | 0.9577 | 0.8700 | 0.1079 |
| *Dendroaspis jamesoni* | Reptile | 0.9909 | 0.9791 | 0.3620 |
| *Dendroaspis polylepis* | Reptile | 0.9117 | 0.7339 | 0.1512 |
| *Dipsadoboa flavida broadleyi* | Reptile | 0.9543 | 0.9156 | 0.1140 |
| *Dispholidus typus* | Reptile | 0.9273 | 0.7775 | 0.1134 |
| *Duberria lutrix* | Reptile | 0.9534 | 0.8817 | 0.2017 |
| *Echis carinatus* | Reptile | 0.9729 | 0.9443 | 0.2820 |
| *Echis pyramidum* | Reptile | 0.9283 | 0.7823 | 0.1583 |
| *Elapsoidea loveridgei* | Reptile | 0.9364 | 0.7731 | 0.1432 |
| *Elapsoidea nigra* | Reptile | 0.9985 | 0.9927 | 0.4217 |
| *Eretmochelys imbricata* | Reptile | 0.9963 | 0.9959 | 0.6094 |
| *Eryx colubrinus* | Reptile | 0.9022 | 0.7591 | 0.1198 |
| *Eumecia anchietae* | Reptile | 0.9743 | 0.9421 | 0.2029 |
| *Gastropholis prasina* | Reptile | 0.9986 | 0.9960 | 0.3064 |
| *Gastropholis vittata* | Reptile | 0.9969 | 0.9938 | 0.5634 |
| *Geocalamus acutus* | Reptile | 0.9744 | 0.9704 | 0.4168 |
| *Gerrhosaurus flavigularis* | Reptile | 0.9015 | 0.7390 | 0.0638 |

**Table S5 Ensemble Model performance of each species studied (continued)**

| **Species** | **Taxonomic Group** | **AUC** | **TSS** | **Threshold** |
| --- | --- | --- | --- | --- |
| *Gerrhosaurus intermedius* | Reptile | 0.9357 | 0.8656 | 0.1962 |
| *Gracililima nyassae* | Reptile | 0.9685 | 0.9536 | 0.3397 |
| *Grayia smithii* | Reptile | 0.9960 | 0.9903 | 0.4319 |
| *Hapsidophrys lineatus* | Reptile | 0.9998 | 0.9996 | 0.7010 |
| *Heliobolus spekii* | Reptile | 0.9190 | 0.7930 | 0.2266 |
| *Hemidactylus angulatus* | Reptile | 0.8673 | 0.7348 | 0.2172 |
| *Hemidactylus barbouri* | Reptile | 0.9612 | 0.9165 | 0.1016 |
| *Hemidactylus brookii* | Reptile | 0.8520 | 0.7470 | 0.5022 |
| *Hemidactylus funaiolii* | Reptile | 0.9760 | 0.9676 | 0.2952 |
| *Hemidactylus isolepis* | Reptile | 0.9445 | 0.8459 | 0.2183 |
| *Hemidactylus mabouia* | Reptile | 0.9215 | 0.7510 | 0.2121 |
| *Hemidactylus macropholis* | Reptile | 0.8984 | 0.7816 | 0.0570 |
| *Hemidactylus modestus* | Reptile | 0.9932 | 0.9895 | 0.2914 |
| *Hemidactylus mrimaensis* | Reptile | 0.9755 | 0.9547 | 0.3215 |
| *Hemidactylus platycephalus* | Reptile | 0.9050 | 0.7080 | 0.0906 |
| *Hemidactylus ruspolii* | Reptile | 0.8717 | 0.7467 | 0.1694 |
| *Hemidactylus squamulatus* | Reptile | 0.8840 | 0.8061 | 0.2168 |
| *Hemirhagerrhis hildebrandtii* | Reptile | 0.9040 | 0.7490 | 0.0380 |
| *Hemirhagerrhis kelleri* | Reptile | 0.8807 | 0.7540 | 0.1270 |
| *Hemirhagerrhis nototaenia* | Reptile | 0.9052 | 0.8386 | 0.2669 |
| *Holodactylus africanus* | Reptile | 0.9687 | 0.9396 | 0.2728 |
| *Homopholis fasciata* | Reptile | 0.8213 | 0.8018 | 0.0122 |
| *Kinixys belliana* | Reptile | 0.9367 | 0.8734 | 0.1319 |
| *Kinixys spekii* | Reptile | 0.9200 | 0.7644 | 0.1224 |
| *Kinixys zombensis* | Reptile | 0.9890 | 0.9744 | 0.3518 |
| *Kinyongia boehmei* | Reptile | 0.9892 | 0.9665 | 0.2397 |
| *Kinyongia excubitor* | Reptile | 0.9607 | 0.8892 | 0.1390 |
| *Kinyongia tavetana* | Reptile | 0.9809 | 0.9499 | 0.3066 |
| *Latastia longicaudata* | Reptile | 0.9290 | 0.7473 | 0.1108 |
| *Leptosiaphos kilimensis* | Reptile | 0.9628 | 0.8923 | 0.1073 |
| *Leptotyphlops keniensis* | Reptile | 0.9851 | 0.9770 | 0.3756 |
| *Leptotyphlops macrops* | Reptile | 0.9475 | 0.9035 | 0.3237 |

**Table S5 Ensemble Model performance of each species studied (continued)**

| **Species** | **Taxonomic Group** | **AUC** | **TSS** | **Threshold** |
| --- | --- | --- | --- | --- |
| *Leptotyphlops merkeri* | Reptile | 0.9324 | 0.8760 | 0.3076 |
| *Leptotyphlops pitmani* | Reptile | 0.9784 | 0.9662 | 0.3778 |
| *Letheobia lumbriciformis* | Reptile | 0.9926 | 0.9852 | 0.3520 |
| *Letheobia mbeerensis* | Reptile | 0.9933 | 0.9915 | 0.5893 |
| *Limaformosa chanleri* | Reptile | 0.9161 | 0.8617 | 0.1308 |
| *Limaformosa savorgnani* | Reptile | 0.9953 | 0.9939 | 0.3597 |
| *Lycophidion capense loveridgei* | Reptile | 0.9897 | 0.9738 | 0.1824 |
| *Lycophidion capense* | Reptile | 0.9048 | 0.7400 | 0.0971 |
| *Lycophidion depressirostre* | Reptile | 0.9137 | 0.7984 | 0.0996 |
| *Lycophidion ornatum* | Reptile | 0.9659 | 0.8965 | 0.1845 |
| *Lygodactylus broadleyi* | Reptile | 0.9899 | 0.9766 | 0.4700 |
| *Lygodactylus capensis* | Reptile | 0.9499 | 0.8682 | 0.1257 |
| *Lygodactylus keniensis* | Reptile | 0.9052 | 0.7735 | 0.1374 |
| *Lygodactylus laterimaculatus* | Reptile | 0.9060 | 0.7627 | 0.1507 |
| *Lygodactylus manni* | Reptile | 0.9433 | 0.9025 | 0.1276 |
| *Lygodactylus mombasicus* | Reptile | 0.9864 | 0.9608 | 0.1866 |
| *Lygodactylus picturatus* | Reptile | 0.8720 | 0.7250 | 0.2479 |
| *Lygodactylus scheffleri* | Reptile | 0.9927 | 0.9904 | 0.3768 |
| *Lygodactylus scorteccii* | Reptile | 0.9373 | 0.8432 | 0.1610 |
| *Lygodactylus somalicus* | Reptile | 0.9331 | 0.9189 | 0.3382 |
| *Lygodactylus tsavoensis* | Reptile | 0.9179 | 0.7845 | 0.1159 |
| *Lygodactylus wojnowskii* | Reptile | 0.9529 | 0.8713 | 0.1620 |
| *Lygosoma sundevalli* | Reptile | 0.9264 | 0.8328 | 0.1083 |
| *Mabuya brevicollis* | Reptile | 0.9254 | 0.8446 | 0.1469 |
| *Mabuya striata* | Reptile | 0.9130 | 0.7535 | 0.0326 |
| *Malacochersus tornieri* | Reptile | 0.9374 | 0.7843 | 0.1567 |
| *Meizodon semiornatus* | Reptile | 0.9237 | 0.7971 | 0.1982 |
| *Melanoseps pygmaeus* | Reptile | 0.9667 | 0.9534 | 0.3381 |
| *Micrelaps bicoloratus* | Reptile | 0.9196 | 0.8217 | 0.1546 |
| *Micrelaps vaillanti* | Reptile | 0.9418 | 0.8628 | 0.2931 |
| *Mochlus sundevallii* | Reptile | 0.9106 | 0.7634 | 0.1968 |
| *Montatheris hindii* | Reptile | 0.9892 | 0.9819 | 0.4444 |

**Table S5 Ensemble Model performance of each species studied (continued)**

| **Species** | **Taxonomic Group** | **AUC** | **TSS** | **Threshold** |
| --- | --- | --- | --- | --- |
| *Naja ashei* | Reptile | 0.9331 | 0.7859 | 0.1543 |
| *Naja haje* | Reptile | 0.9540 | 0.8698 | 0.1420 |
| *Naja nigricollis* | Reptile | 0.9185 | 0.7600 | 0.1305 |
| *Naja pallida* | Reptile | 0.9170 | 0.7268 | 0.1285 |
| *Naja subfulva* | Reptile | 0.9486 | 0.8348 | 0.1133 |
| *Natriciteres olivacea* | Reptile | 0.9111 | 0.8245 | 0.2059 |
| *Nucras boulengeri* | Reptile | 0.9488 | 0.8660 | 0.1395 |
| *Panaspis massaiensis* | Reptile | 0.9338 | 0.7947 | 0.1664 |
| *Panaspis tsavoensis* | Reptile | 0.9528 | 0.9143 | 0.3627 |
| *Panaspis wahlbergii* | Reptile | 0.9212 | 0.8322 | 0.2936 |
| *Pelomedusa neumanni* | Reptile | 0.8920 | 0.7883 | 0.0755 |
| *Pelomedusa subrufa* | Reptile | 0.8920 | 0.7499 | 0.2091 |
| *Pelusios sinuatus* | Reptile | 0.9610 | 0.8768 | 0.1335 |
| *Pelusios williamsi* | Reptile | 0.9760 | 0.9760 | 0.5045 |
| *Philochortus rudolfensis* | Reptile | 0.9448 | 0.9095 | 0.2245 |
| *Philothamnus battersbyi* | Reptile | 0.9253 | 0.7599 | 0.1329 |
| *Philothamnus carinatus* | Reptile | 0.9943 | 0.9935 | 0.4021 |
| *Philothamnus heterolepidotus* | Reptile | 0.9745 | 0.9683 | 0.2853 |
| *Philothamnus hoplogaster* | Reptile | 0.9480 | 0.8751 | 0.0769 |
| *Philothamnus irregularis* | Reptile | 0.9279 | 0.8701 | 0.3646 |
| *Philothamnus macrops* | Reptile | 0.9934 | 0.9892 | 0.2104 |
| *Philothamnus nitidus loveridgei* | Reptile | 0.9909 | 0.9793 | 0.4019 |
| *Philothamnus punctatus* | Reptile | 0.9424 | 0.8425 | 0.1339 |
| *Philothamnus semivariegatus* | Reptile | 0.9038 | 0.7449 | 0.1628 |
| *Platyceps brevis* | Reptile | 0.9029 | 0.7790 | 0.1607 |
| *Platyceps florulentus* | Reptile | 0.9289 | 0.9112 | 0.1833 |
| *Polemon christyi* | Reptile | 0.9966 | 0.9962 | 0.4921 |
| *Prosymna ruspolii* | Reptile | 0.9041 | 0.8372 | 0.0378 |
| *Prosymna stuhlmanni* | Reptile | 0.9161 | 0.8080 | 0.1451 |
| *Psammophis biseriatus* | Reptile | 0.9430 | 0.7970 | 0.1090 |
| *Psammophis lineatus* | Reptile | 0.9917 | 0.9843 | 0.3193 |
| *Psammophis mossambicus* | Reptile | 0.9257 | 0.7280 | 0.0803 |

**Table S5 Ensemble Model performance of each species studied (continued)**

| **Species** | **Taxonomic Group** | **AUC** | **TSS** | **Threshold** |
| --- | --- | --- | --- | --- |
| *Psammophis orientalis* | Reptile | 0.9825 | 0.9646 | 0.2730 |
| *Psammophis phillipsii* | Reptile | 0.9553 | 0.9542 | 0.1951 |
| *Psammophis pulcher* | Reptile | 0.9580 | 0.9508 | 0.2921 |
| *Psammophis punctulatus* | Reptile | 0.8600 | 0.7030 | 0.4574 |
| *Psammophis sudanensis* | Reptile | 0.9359 | 0.7956 | 0.2003 |
| *Psammophis tanganicus* | Reptile | 0.9271 | 0.8064 | 0.1355 |
| *Psammophylax multisquamis* | Reptile | 0.9479 | 0.8689 | 0.1354 |
| *Pseudaspis cana* | Reptile | 0.9716 | 0.9278 | 0.1049 |
| *Pseuderemias smithii* | Reptile | 0.9171 | 0.8481 | 0.1448 |
| *Python natalensis* | Reptile | 0.9370 | 0.8336 | 0.1590 |
| *Python sebae* | Reptile | 0.9239 | 0.7885 | 0.1907 |
| *Rhamnophis aethiopissa elgonensis* | Reptile | 0.9829 | 0.9708 | 0.3636 |
| *Rhamphiophis oxyrhynchus* | Reptile | 0.8966 | 0.8221 | 0.1732 |
| *Rhamphiophis rostratus* | Reptile | 0.9430 | 0.7840 | 0.1667 |
| *Rhamphiophis rubropunctatus* | Reptile | 0.9105 | 0.7515 | 0.1040 |
| *Rhinotyphlops unitaeniatus* | Reptile | 0.8746 | 0.7382 | 0.0921 |
| *Rieppeleon kerstenii* | Reptile | 0.9477 | 0.8852 | 0.1759 |
| *Scaphiophis albopunctatus* | Reptile | 0.9349 | 0.8486 | 0.1428 |
| *Scaphiophis raffreyi* | Reptile | 0.9620 | 0.9443 | 0.2277 |
| *Stenodactylus sthenodactylus* | Reptile | 0.9559 | 0.9239 | 0.3364 |
| *Stigmochelys pardalis* | Reptile | 0.9313 | 0.7607 | 0.2664 |
| *Telescopus dhara somalicus* | Reptile | 0.9319 | 0.7925 | 0.1868 |
| *Telescopus obtusus* | Reptile | 0.9291 | 0.8358 | 0.1091 |
| *Telescopus semiannulatus* | Reptile | 0.9346 | 0.8288 | 0.1410 |
| *Thelotornis mossambicanus* | Reptile | 0.9454 | 0.8515 | 0.0721 |
| *Thelotornis usambaricus* | Reptile | 0.9856 | 0.9680 | 0.3012 |
| *Thrasops jacksonii* | Reptile | 0.9473 | 0.8667 | 0.1596 |
| *Thrasops schmidti* | Reptile | 0.9802 | 0.9665 | 0.2542 |
| *Toxicodryas adamanteus* | Reptile | 0.9863 | 0.9820 | 0.3577 |
| *Toxicodryas vexator* | Reptile | 0.9991 | 0.9988 | 0.6190 |
| *Trachylepis bayonii* | Reptile | 0.9773 | 0.9344 | 0.1957 |

**Table S5 Ensemble Model performance of each species studied (continued)**

| **Species** | **Taxonomic Group** | **AUC** | **TSS** | **Threshold** |
| --- | --- | --- | --- | --- |
| *Trachylepis brevicollis* | Reptile | 0.8975 | 0.7130 | 0.1345 |
| *Trachylepis dichroma* | Reptile | 0.9653 | 0.9395 | 0.2301 |
| *Trachylepis irregularis* | Reptile | 0.9684 | 0.9506 | 0.1312 |
| *Trachylepis maculilabris* | Reptile | 0.9668 | 0.9004 | 0.1731 |
| *Trachylepis margaritifera* | Reptile | 0.9738 | 0.9165 | 0.1076 |
| *Trachylepis megalura* | Reptile | 0.9400 | 0.8128 | 0.1057 |
| *Trachylepis planifrons* | Reptile | 0.9289 | 0.7986 | 0.1298 |
| *Trachylepis quinquetaeniata* | Reptile | 0.9289 | 0.7813 | 0.2000 |
| *Trachylepis striata* | Reptile | 0.9028 | 0.7533 | 0.1816 |
| *Trachylepis varia* | Reptile | 0.9457 | 0.8237 | 0.1078 |
| *Trioceros bitaeniatus* | Reptile | 0.9505 | 0.8348 | 0.1299 |
| *Trioceros ellioti* | Reptile | 0.9475 | 0.8920 | 0.0869 |
| *Trioceros hoehnelii* | Reptile | 0.9666 | 0.8626 | 0.1924 |
| *Trioceros jacksonii jacksonii* | Reptile | 0.9818 | 0.9594 | 0.2076 |
| *Trioceros jacksonii xantholophus* | Reptile | 0.9783 | 0.9376 | 0.2949 |
| *Trioceros jacksonii* | Reptile | 0.9701 | 0.8916 | 0.1100 |
| *Trioceros kinangopensis* | Reptile | 0.9919 | 0.9905 | 0.3069 |
| *Trioceros schubotzi* | Reptile | 0.9642 | 0.9340 | 0.3266 |
| *Trionyx triunguis* | Reptile | 0.9520 | 0.8944 | 0.1330 |
| *Varanus albigularis* | Reptile | 0.9360 | 0.7843 | 0.1999 |
| *Varanus exanthematicus* | Reptile | 0.9345 | 0.9260 | 0.2422 |
| *Varanus niloticus* | Reptile | 0.9221 | 0.7763 | 0.2217 |


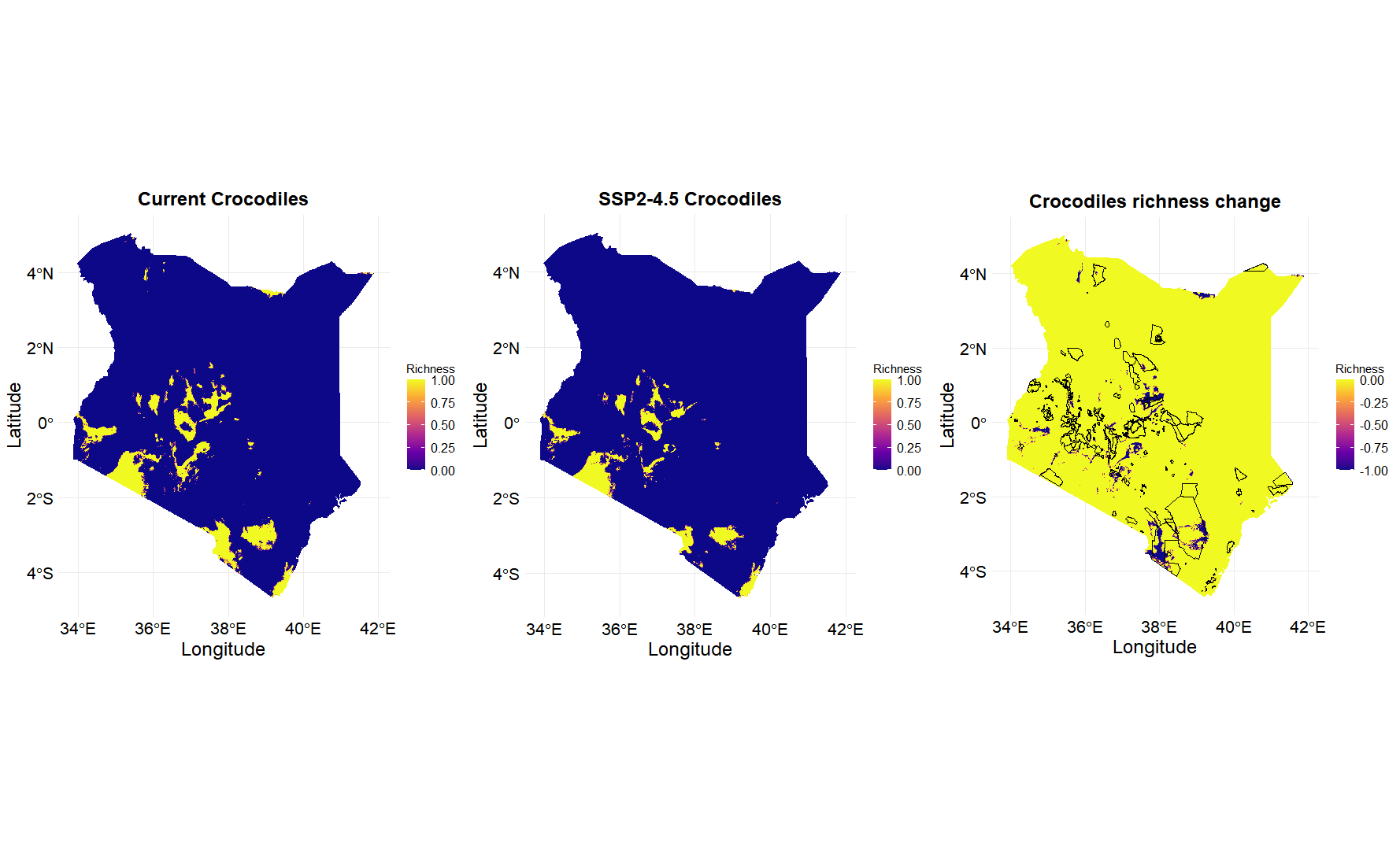


**Figure S14 shows the Richness of Crocodile species in Kenya for current and SSP2-4.5 climate change scenario and the richness change.**

**NB:** As only one crocodile species is native to Kenya, richness is equivalent to the presence (1) or absence (0) of *Crocodylus niloticus*. Projected richness change shows areas of potential local extinction (-1)


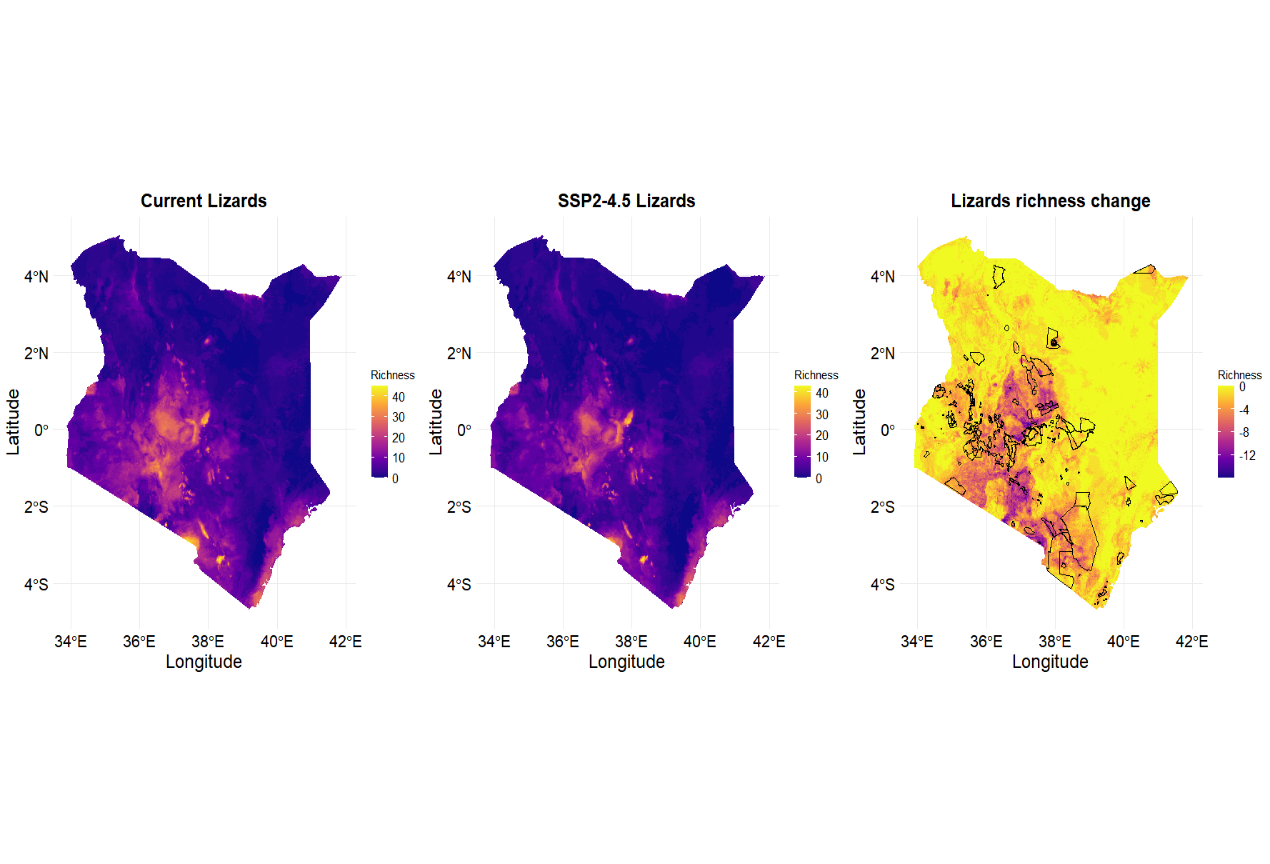


**Figure S15 shows the Richness of Lizard species in Kenya for current and SSP2-4.5 climate change scenario and the richness change.**


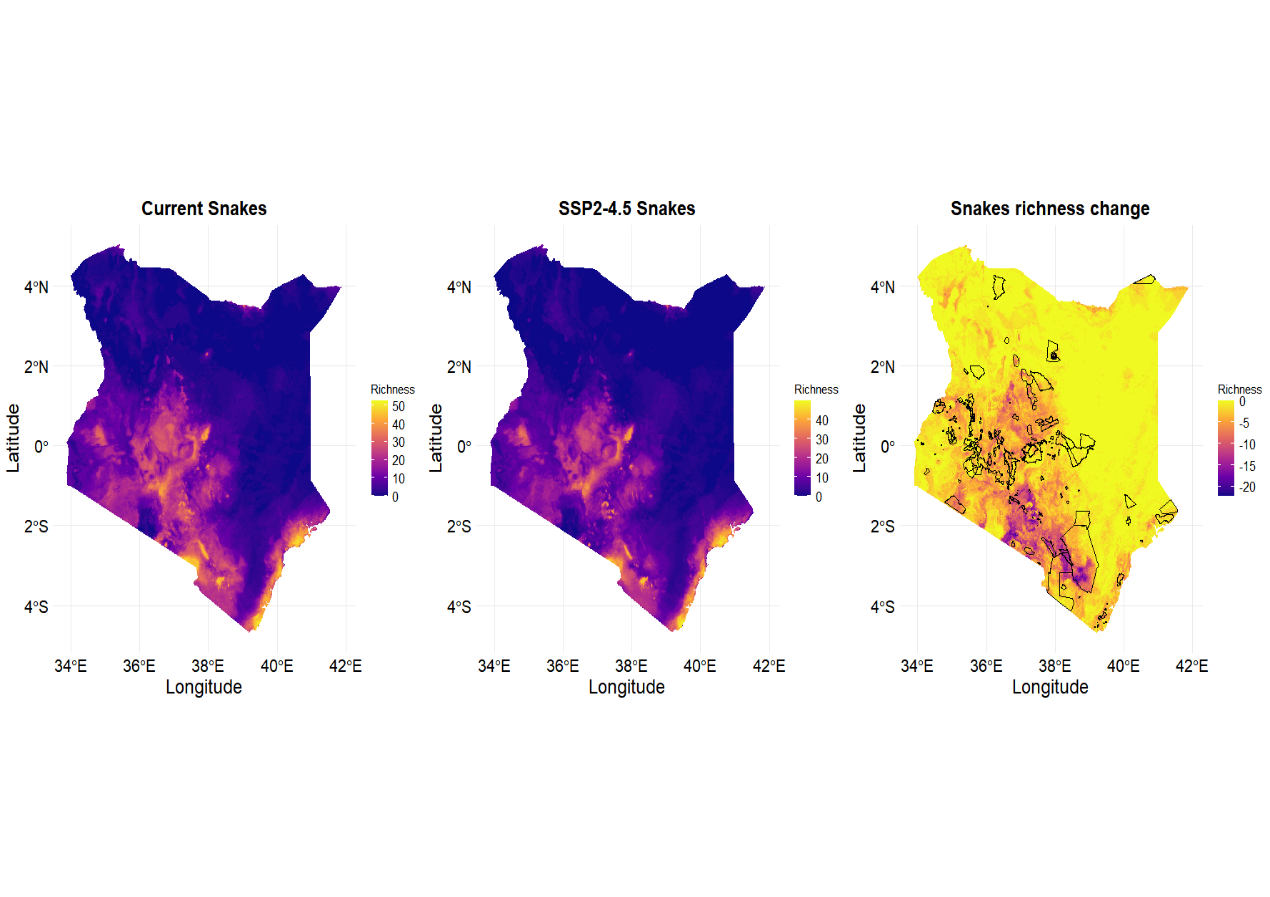


**Figure S16 shows the Richness of Snake species in Kenya for current and SSP2-4.5 climate change scenario and the richness change.**


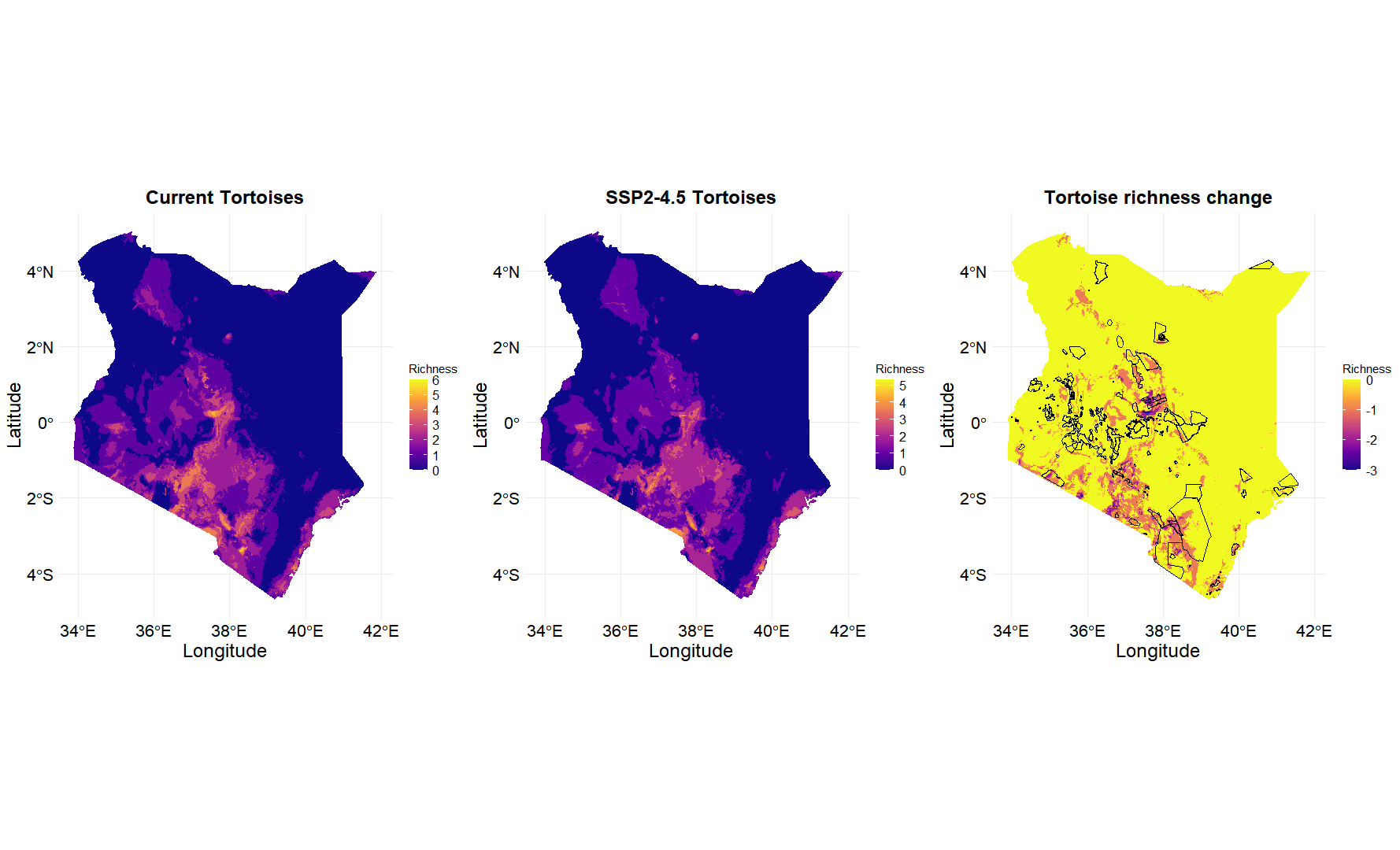


**Figure S17 shows the Richness of Tortoise species in Kenya for current and SSP2-4.5 climate change scenario and the richness change.**


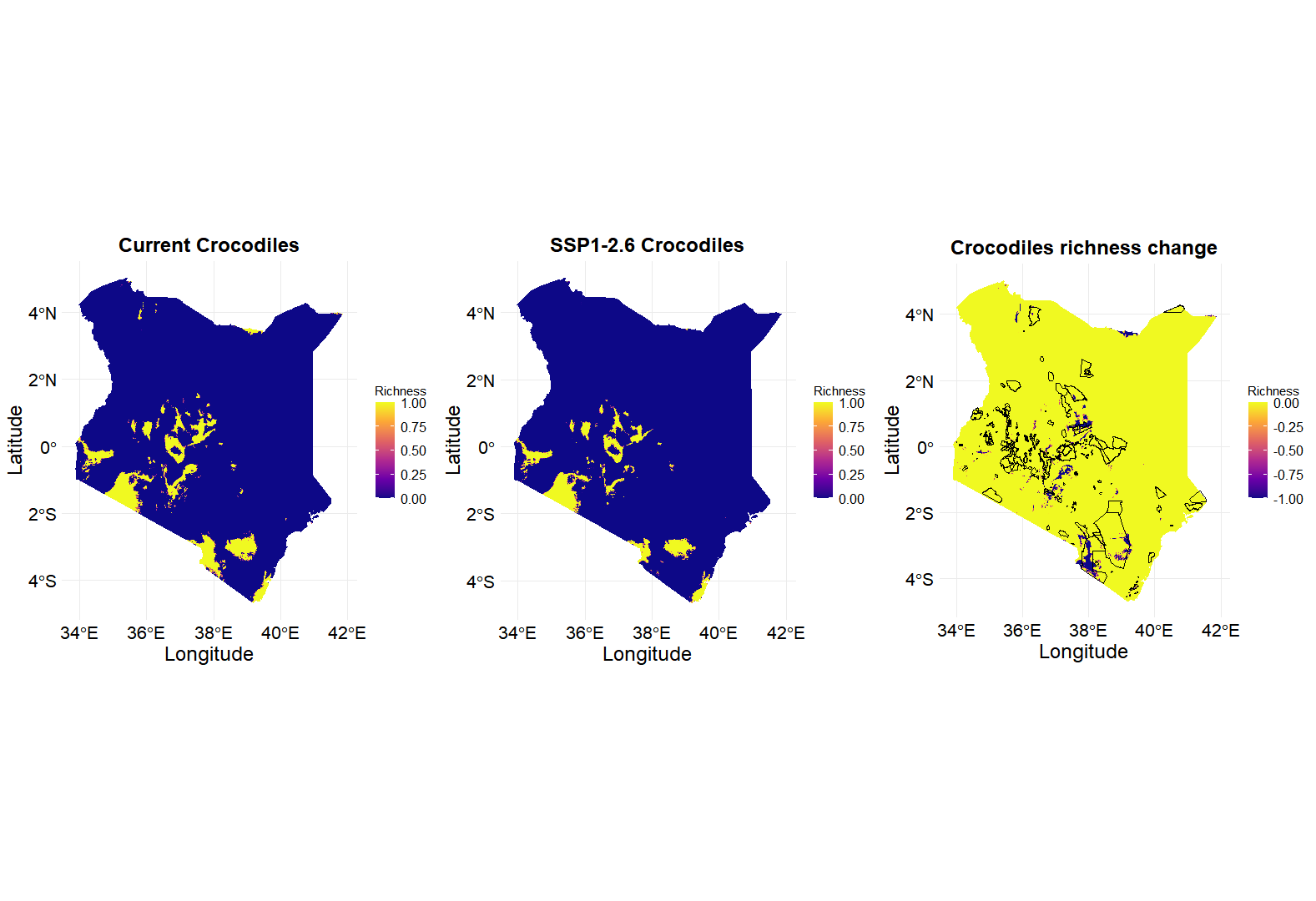


**Figure S18 shows the Richness of Crocodile species in Kenya for current and SSP1-2.6 climate change scenario and the richness change.**

**NB:** As only one crocodile species is native to Kenya, richness is equivalent to the presence (1) or absence (0) of *Crocodylus niloticus*. Projected richness change shows areas of potential local extinction (-1)


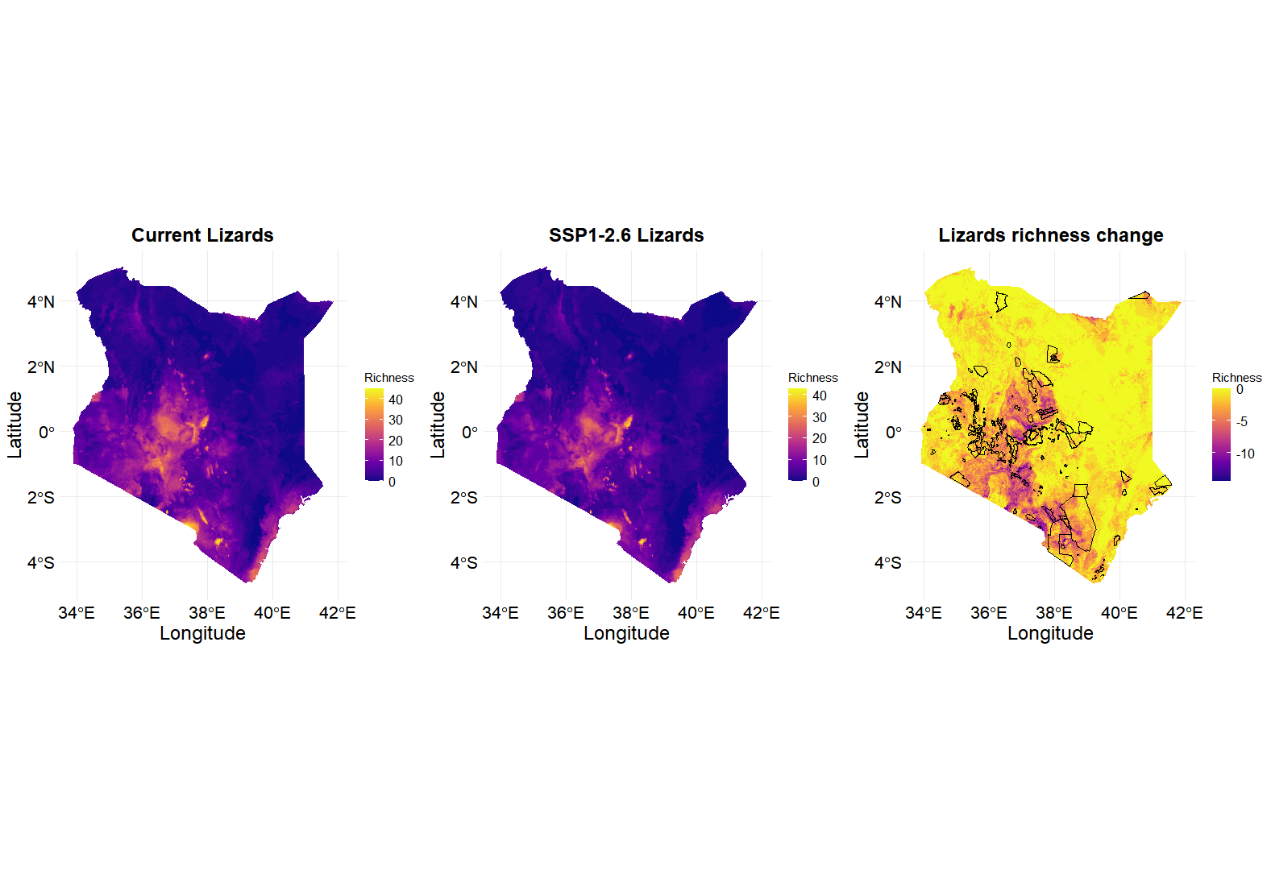


**Figure S19 shows the Richness of Lizard species in Kenya for the current and SSP1-2.6 climate change scenario and the richness change.**


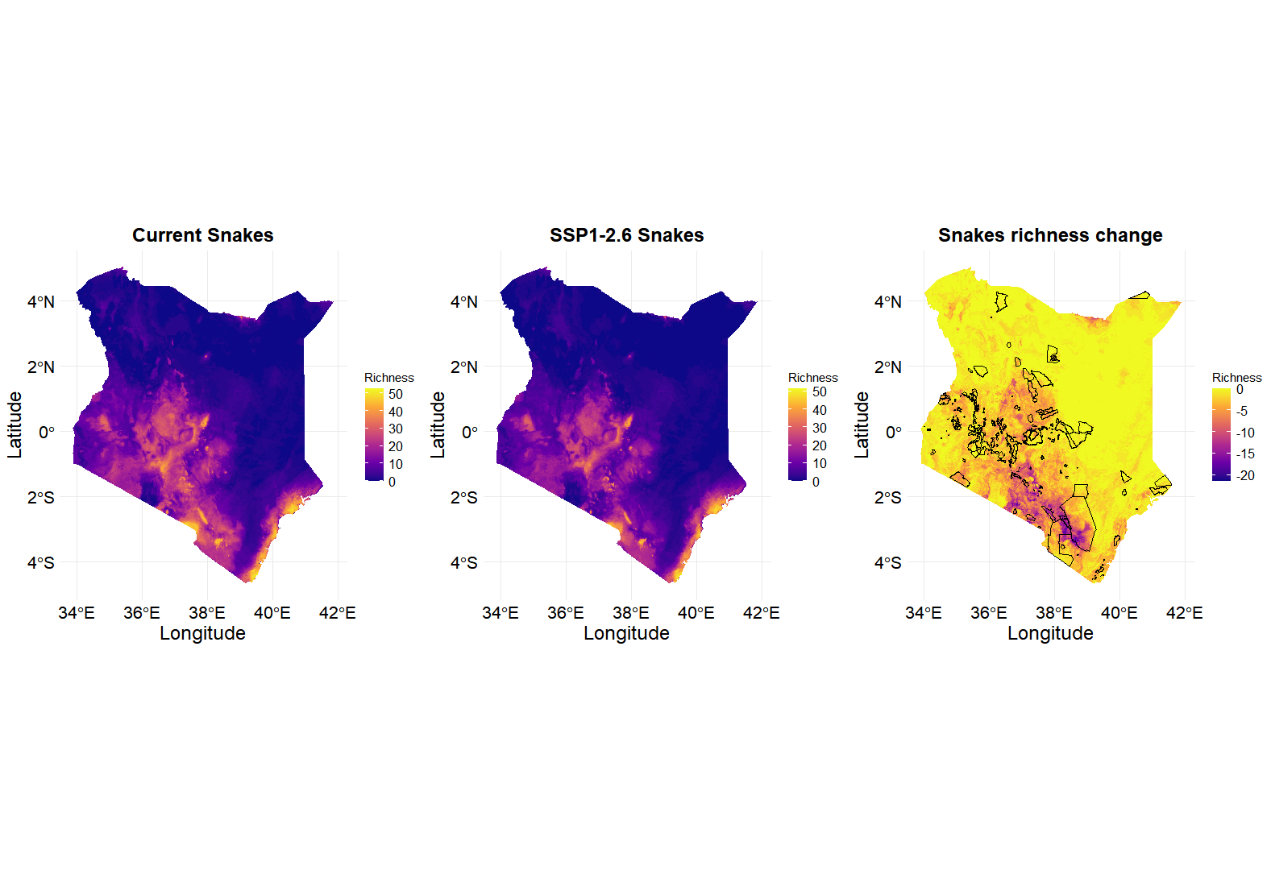


**Figure S20 shows the Richness of Snakes species in Kenya for the current and SSP1-2.6 climate change scenarios and the richness change.**


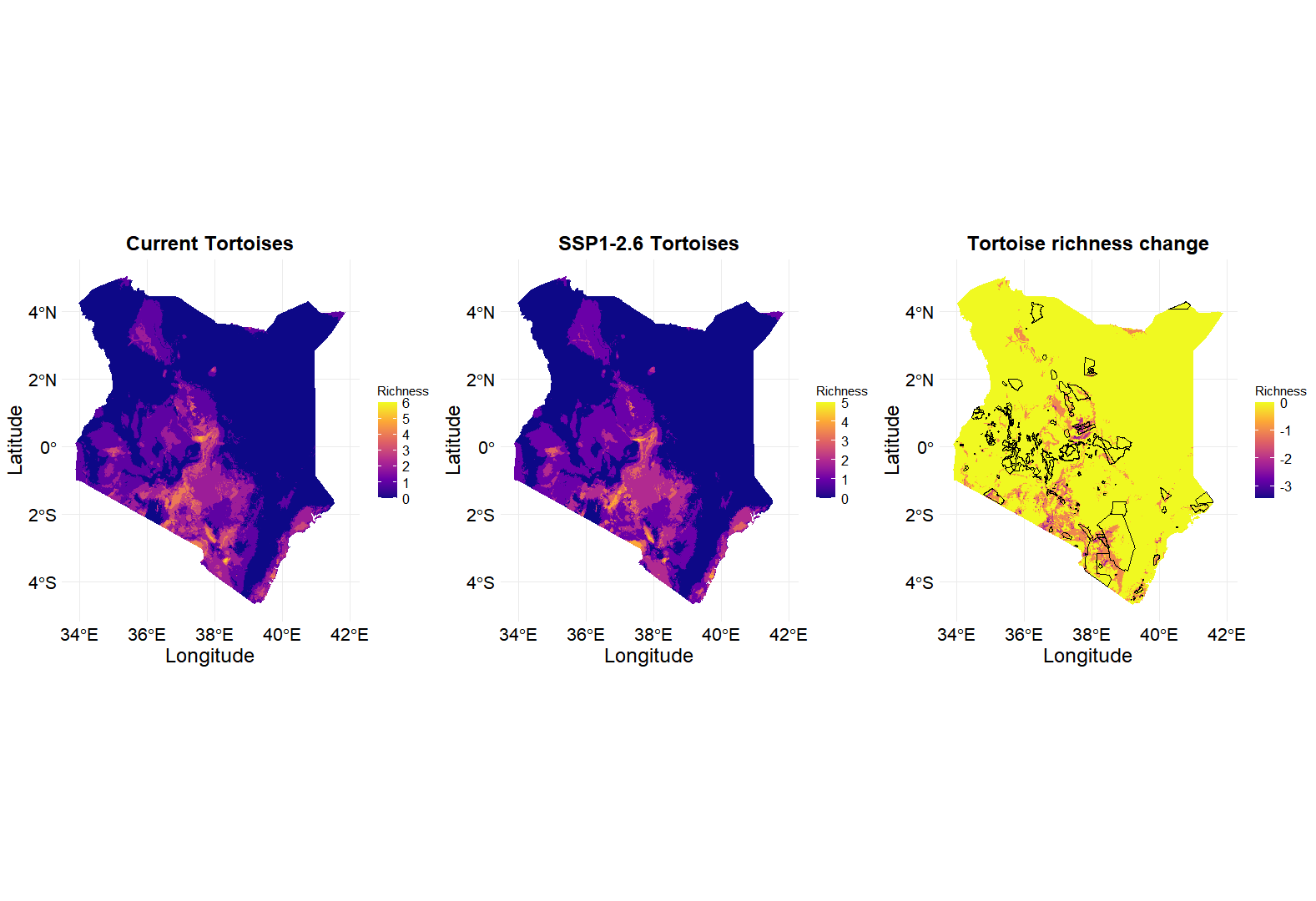


**Figure S21 shows the Richness of Tortoise species in Kenya for the current and SSP1-2.6 climate change scenarios and the richness change.**


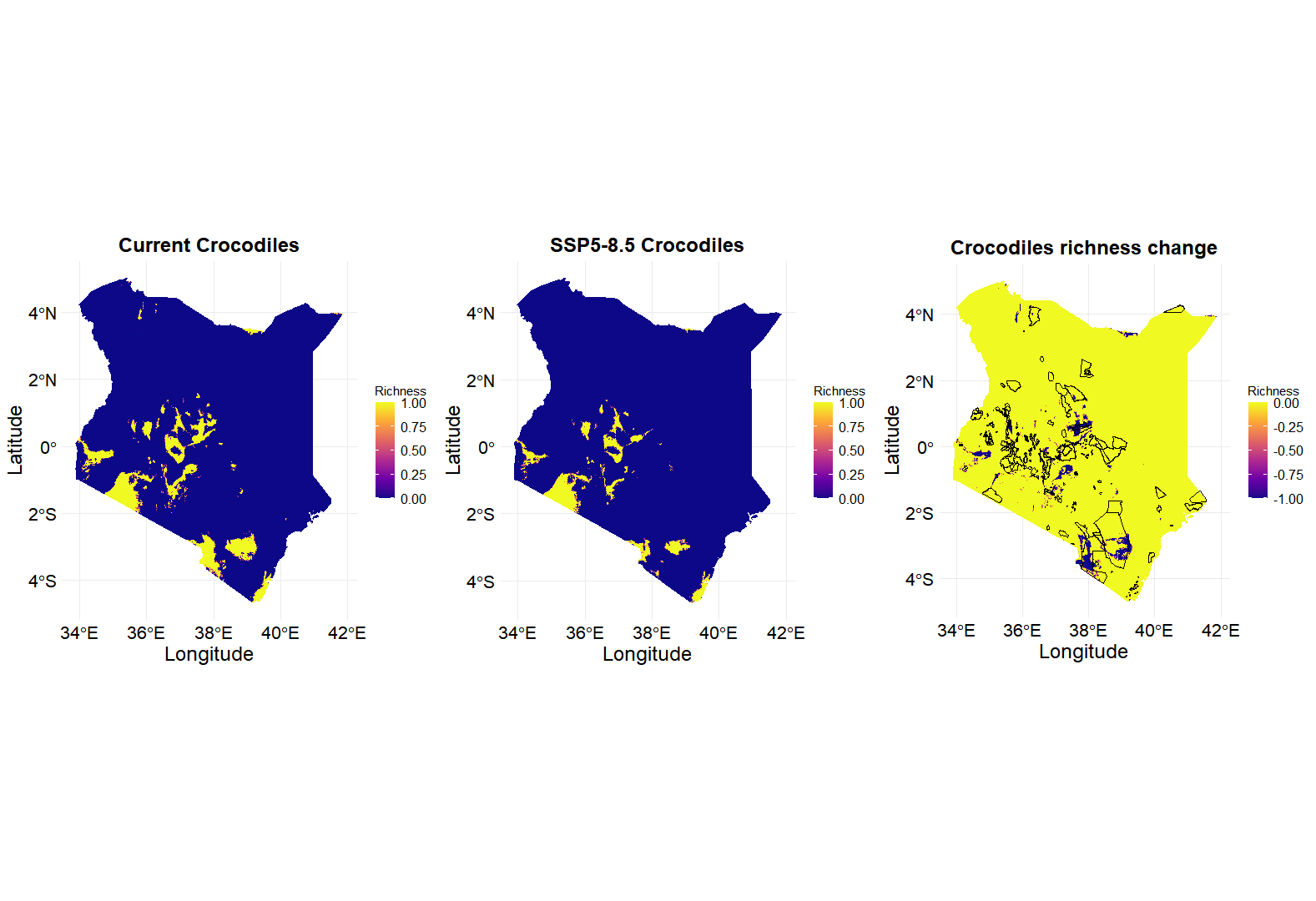


**Figure S22 shows the Richness of Crocodile species in Kenya for the current and SSP5-8.5 climate change scenarios and the richness change.**

**NB:** As only one crocodile species is native to Kenya, richness is equivalent to the presence (1) or absence (0) of *Crocodylus niloticus*. Projected richness change shows areas of potential local extinction (-1)


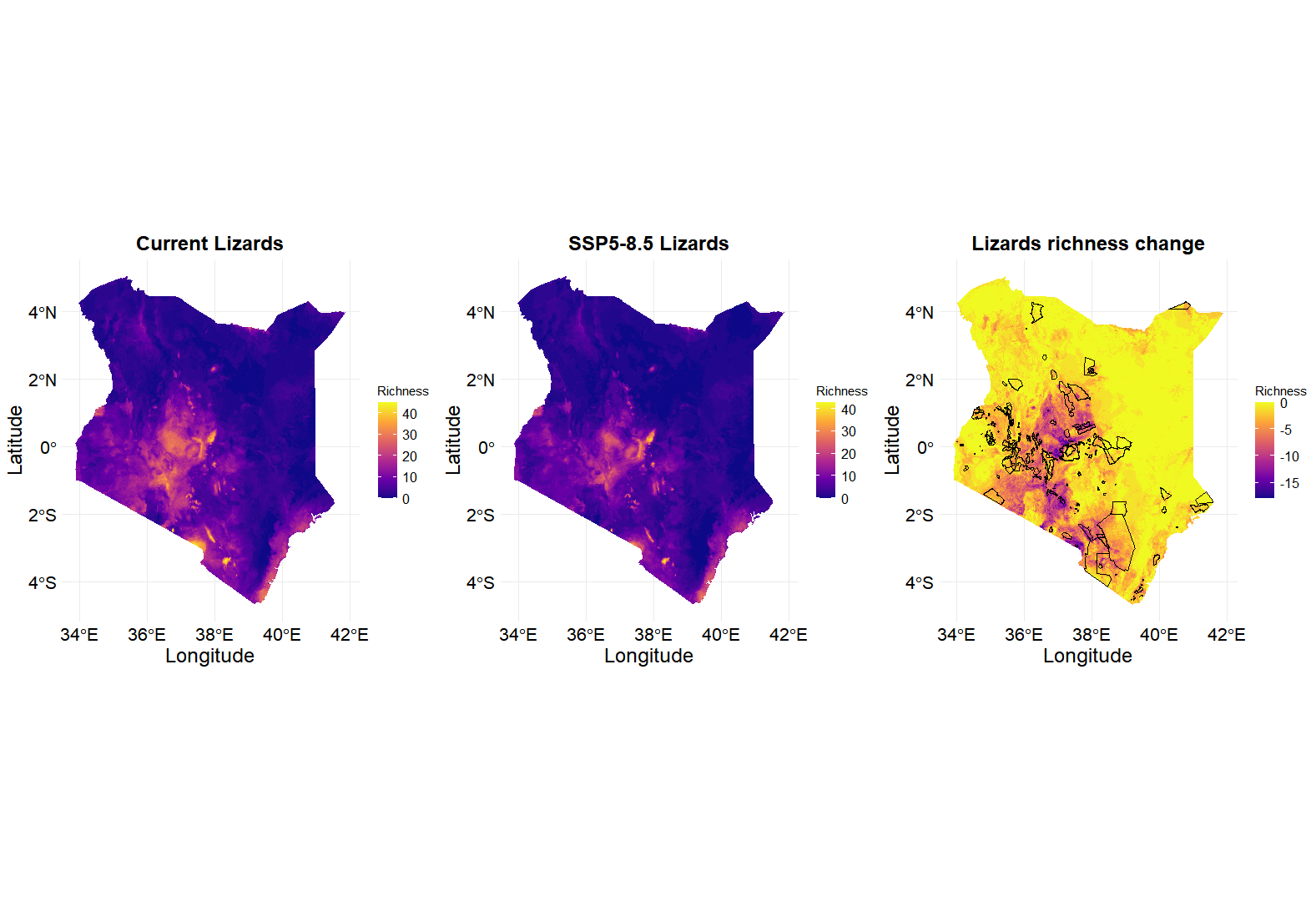


**Figure S23 shows the Richness of Lizard species in Kenya for the current and SSP5-8.5 climate change scenarios and the richness change.**


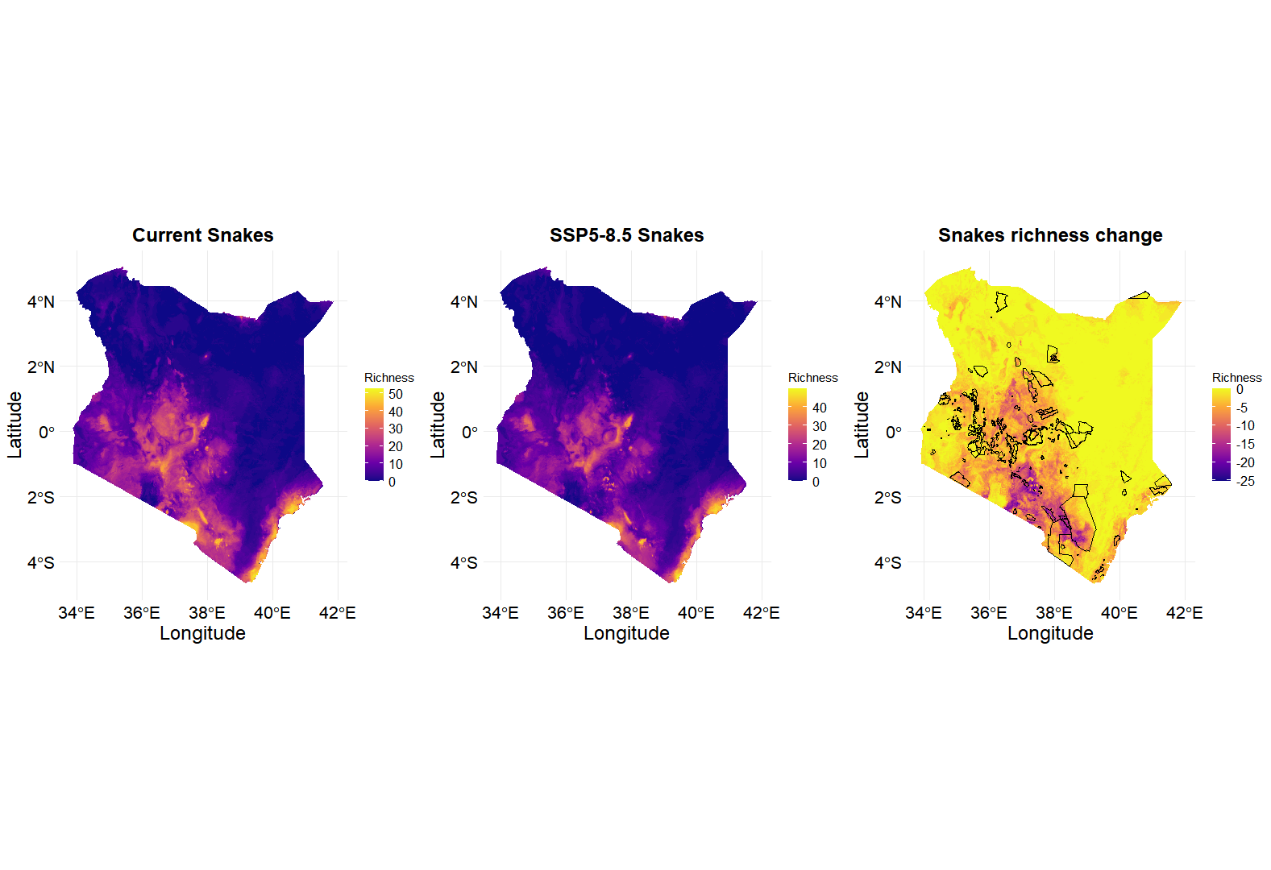


**Figure S24 shows the Richness of Lizard species in Kenya for the current and SSP5-8.5 climate change scenarios and the richness change.**


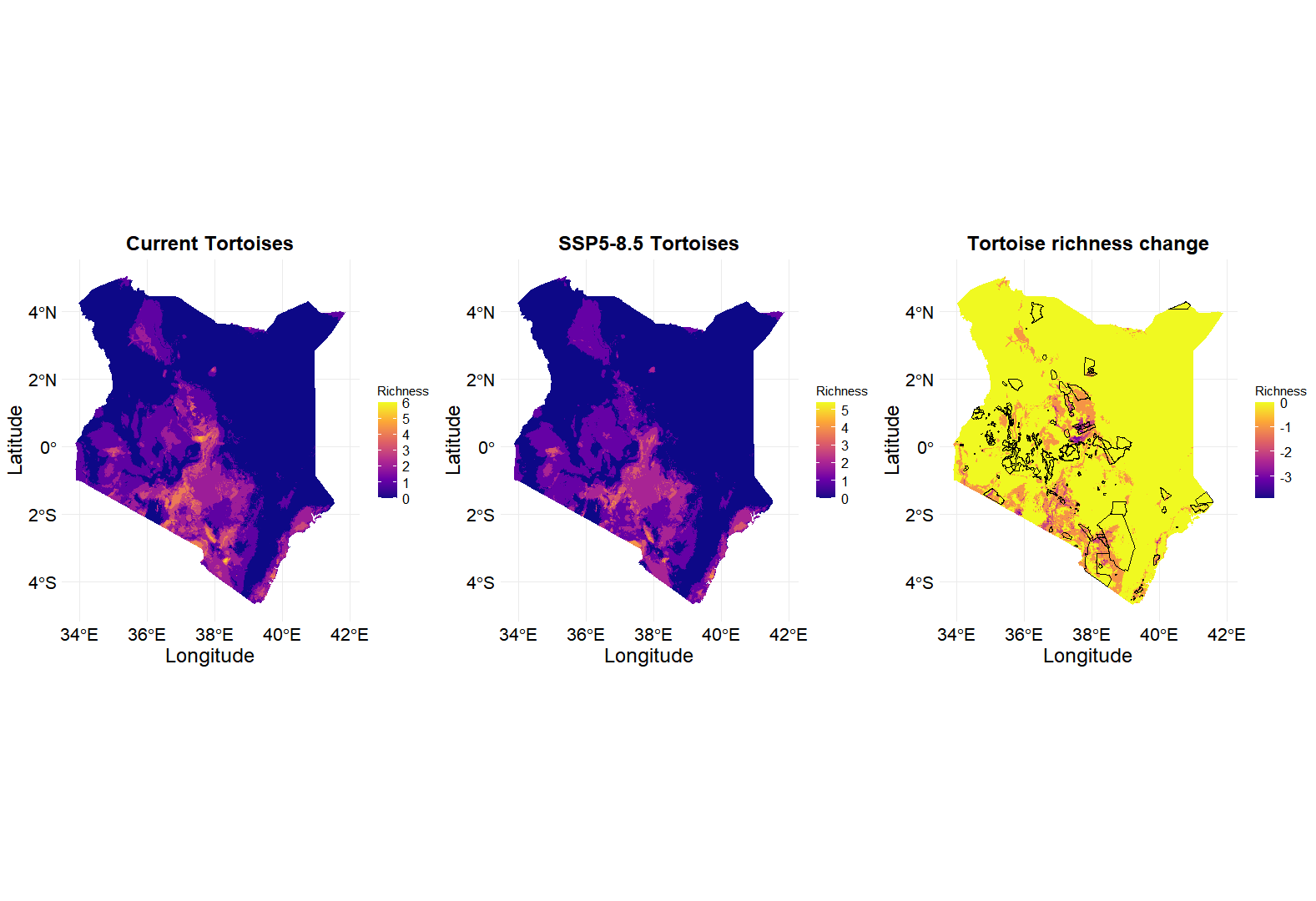


**Figure S25 shows the Richness of Tortoise species in Kenya for the current and SSP5-8.5 climate change scenarios and the richness change.**
